# Assessing paedophilic sexual preferences in child sexual offenders using eye-tracking methods: an exploratory meta-analysis

**DOI:** 10.3389/fpsyg.2026.1908178

**Published:** 2026-09-08

**Authors:** Jenthe Mens, Erik Masthoff, Pauline Heus, Stefan Bogaerts

**Affiliations:** 1 Department of Developmental Psychology, Tilburg University, Tilburg, Netherlands; 2 Fivoor Science and Treatment Innovation, Fivoor, Rotterdam, Netherlands; 3 Cochrane Netherlands, University Medical Center Utrecht, Utrecht University, Utrecht, Netherlands; 4 Julius Center for Health Sciences and Primary Care, University Medical Center Utrecht, Utrecht University, Utrecht, Netherlands

**Keywords:** child sexual offending, exploratory meta-analysis, eye-tracking, forensic psychiatry, paedophilia

## Abstract

Due to reliability issues associated with self-reported sexual interest in children among individuals with paedophilia convicted of child sexual offences (PCSO), more innovative assessment methods have been studied for years. One such method involves eye-tracking techniques, which can provide insights into (automatic) attentional processes that may reflect underlying deviant sexual preferences. The current exploratory meta-analysis aimed to evaluate whether eye-tracking measurements are associated with paedophilic sexual preferences by examining group-level differences between diagnosed individuals and comparison groups. A systematic literature search was conducted in PubMed, PsycINFO, Embase, Web of Science, Scopus, Cochrane Library, and Google Scholar, up to January 18, 2026. Ten relevant publications were found. Due to data overlap, only five studies were included, yielding 12 effect sizes. No significant group differences were found for viewing time. However, PCSOs fixated faster on child stimuli compared to individuals convicted of adult sex offences, offenders, and individuals without clinical signs of paedophilia. Additionally, the results provided evidence that PCSOs preferred viewing child stimuli over adult stimuli. It is concluded that, although the results should be interpreted with caution, there is some evidence that eye-tracking techniques can be useful for distinguishing differences in paedophilic sexual preferences. However, more empirical research is needed to determine the value of eye-tracking techniques for clinical practice.

## 1 Introduction

Child sexual offences are high-impact crimes due to the often serious short- and long-term consequences for victims, their immediate environment, and the broader sense of safety in society (Papalia et al., 2020; Yang, 2022). The global prevalence of child sexual abuse is substantial. According to the World Health Organization (2024), approximately one in five women and one in seven men reported having experienced sexual abuse during childhood. Furthermore, a recent systematic review by Fang et al. (2026) estimated that in 2024, approximately 72.6 million girls and 60.9 million boys experienced some form of sexual violence. This included 43.3 million girls and 34.8 million boys who were victims of rape or sexual assault. Ali et al. (2024) further emphasised that child sexual abuse occurs across all social classes, races, ethnicities, and in every historical era. These alarming prevalence numbers highlight the need to develop and implement more innovative assessment and monitoring methods to improve the identification of risk factors associated with child sexual abuse and to support more effective prevention strategies.

Although research indicates that only approximately 10 to 50% of men who have sexually offended against children meet the diagnostic criteria for paedophilic disorder (Gerwinn et al., 2018; Gouveia et al., 2024; Schmidt et al., 2013; Seto, 2009, 2018, 2019), paedophilia has consistently been identified as a robust risk factor for sexual recidivism among individuals who have sexually offended against children (Hanson and Bussière, 1998; Hanson and Morton-Bourgon, 2005; Seto, 2018). Consequently, the assessment of sexual attraction to prepubescent children represents a key component of comprehensive clinical risk assessment. In clinical settings, individuals with paedophilic sexual interests may be diagnosed with paedophilic disorder if they meet the diagnostic criteria outlined in the Diagnostic and Statistical Manual of Mental Disorders (American Psychiatric Association, 2022). This disorder is characterised by recurrent and intense sexually arousing fantasies, urges, or behaviours involving sexual activity with prepubescent children. A diagnosis requires that the individual has acted on these urges or experiences clinically significant distress or interpersonal impairment as a result of them (American Psychiatric Association, 2022). Therefore, the accurate assessment of paedophilic sexual interest, alongside other empirically supported risk factors, is essential for understanding child sexual offending behaviour and informing clinical decision-making.

Conventional assessment and treatment monitoring of paedophilic sexual interest typically rely on a multimethod approach (Seto, 2008). This includes clinical interviews based on the diagnostic criteria for paedophilic disorder (American Psychiatric Association, 2022), self-report questionnaires which provide information on sexual interests, attitudes, and behaviours such as the Multiphasic Sex Inventory (MSI-II; Nichols and Molinder, 2000), and structured assessment instruments such as the Screening Scale for Pedophilic Interests (SSPI-2; Seto et al., 2015) and Correlates of Admitted Sexual Interest in Children Scale (CASIC; Seto and Eke, 2017). Although these assessment approaches provide valuable information, there are some limitations. For example, responses may be influenced by social desirability bias (Tan and Grace, 2008). Individuals who have sexually offended against children may minimise or deny their sexual interest in children (Vásquez-Amézquita et al., 2023). This raises concerns regarding the reliability and validity of current assessment, evaluation, and monitoring tools addressing risk factors of child sexual (re-)offending.

To develop additional innovative assessment tools, researchers have explored physiological biomarkers that are assumed to indirectly index (paedophilic) sexual arousal. In North America, the most established technique is penile plethysmography, which records genital circumference or volume while the subject views or hears sexually suggestive material (Bickle et al., 2021). Plethysmography is criticised for non-standardised protocols, susceptibility to deliberate suppression, and ethical concerns related to its intrusiveness and the use of pornographic stimuli depicting minors (Coric et al., 2005; Sandland and Balmer, 2012). Another approach is polygraphy (‘lie detection’), which measures peripheral physiological changes in sympathetic activity, such as heart rate and skin conductance, during confrontational interviews (Sandland and Balmer, 2012; Buschman et al., 2010). However, the use of polygraphy in forensic psychiatry and legal contexts remains controversial due to concerns regarding its reliability and validity. Functional brain imaging provides a third route: fMRI studies measure regional brain activity while participants view sexual stimuli, revealing discriminative brain patterns even when non-explicit sexual stimuli are presented (Jordan et al., 2015; Jordan et al., 2020; Ponseti et al., 2016). However, neuroimaging remains expensive, time-consuming, uncomfortable, and potentially stressful (Graham and Friesen, 2019). Consequently, there is growing interest in less-intrusive, scalable tools, such as eye-tracking, that can provide indirect indicators of sexual interest in clinical and forensic settings.

### 1.1 Eye-tracking

Eye-tracking unobtrusively measures where and for how long individuals fixate on different parts of an image or video. This technique has been suggested as an indirect measure of (paedophilic) sexual interest because attentional responses toward sexually relevant stimuli may provide information about underlying sexual preferences (Vásquez-Amézquita et al., 2023). In contrast to self-report-based measures, eye-tracking captures behavioural indicators of visual attention that are less dependent on conscious disclosure. With this method, (non-)erotic visual stimuli are presented while the eye movements of the observer (i.e., the individual being studied) are recorded using an infrared camera. These eye movements include fixations (gaze clusters) and saccades (rapid shifts between fixations). More specifically, eye-tracking can determine which stimuli, or specific parts of them (areas of interest), attract visual attention, as well as the time to first fixation (‘entry time’ or ‘fixation latency’) and the total viewing time (measured in milliseconds). The number of fixations and the relative fixation time (i.e., the total duration of all fixations within a particular area of interest divided by total stimulus presentation time) can also be examined (Krajbich et al., 2010).

According to Vásquez-Amézquita et al. (2023), this methodology offers insights into visual attentional processes, as eye movements reveal overt attention directed toward relevant stimuli. The information-processing approach proposed by Spiering and Everaerd (2007) indicates that sexually relevant features of a stimulus are selected pre-attentively, with focal attention automatically directed toward them. Empirical research supports this idea, demonstrating that individuals tend to orient more quickly and maintain their gaze longer on stimuli that align with their sexual interests (Fromberger et al., 2012; Vásquez-Amézquita et al., 2018). Within this context, two distinct attentional processes that may be associated with sexual interests can be distinguished using eye-tracking: initial orienting, typically measured by fixation latency, and sustained attention, measured by fixation duration (Vásquez-Amézquita et al., 2023). Previous research has shown that individuals with a paedophilic disorder exhibit shorter fixation latencies and longer fixation times when viewing child stimuli than forensic and healthy controls (Fromberger et al., 2012). Eye-tracking techniques offer several advantages: they are low-cost, non-invasive, and easy to implement (Vásquez-Amézquita et al., 2023). Furthermore, eye-tracking captures aspects of visual attention that may be less influenced by conscious reporting compared with self-report measures, making the method less vulnerable to deliberate manipulation (Godet and Niveau, 2021; Nummenmaa et al., 2006; Vásquez-Amézquita et al., 2023).

Godet and Niveau (2021) conducted a systematic review to explore the use of eye-tracking techniques as a forensic tool to assess paedophilic sexual interests in individuals convicted of child sexual offences. Their review included six studies, five of which originated from the same research project and involved largely overlapping study populations, study designs, and data. Included studies examined the sexual preferences of paedophilic individuals convicted of child sexual offences through eye-tracking and compared the eye movement data with the results of various control groups. All six studies were presented descriptively, and the review did not include a meta-analysis. The authors suggested that eye-tracking can distinguish between paedophilic and non-paedophilic sexual interests, but that, due to the limited number of studies, more research is needed to establish the usefulness of eye-tracking techniques in the context of forensic psychiatry and criminal law. Schippers et al. (2023) performed a systematic review and several meta-analyses to analyse sexual interest in both children and adults in paedophilic male samples versus comparison groups. Although a few included studies employed eye-tracking, no specific meta-analyses were conducted on this data, as most studies used alternative assessment tools. Lastly, a theoretical review by Vásquez-Amézquita et al. (2023) investigated how conceptual models of sexual information processing support the use of attentional paradigms, such as eye-tracking, as indirect measures of sexual preferences, such as paedophilia. They argued that eye-tracking may, in the future, help differentiate between individuals convicted of sexual offences who have paedophilic sexual interests and those without, and assist in monitoring cognitive-behavioural treatment aimed at improving attentional control over arousal. However, the authors also noted that current psychometric evidence remains inconclusive and that further research is needed (Vásquez-Amézquita et al., 2023). Therefore, despite promising findings, the ability of eye-tracking to differentiate paedophilic sexual interests remains uncertain. A quantitative and qualitative synthesis of the available evidence is needed to determine whether specific eye-tracking measures consistently differentiate individuals with paedophilic sexual interests from relevant comparison groups.

The present exploratory meta-analysis aimed to evaluate whether eye-tracking measurements are associated with paedophilic sexual preferences by examining group-level differences between individuals diagnosed with paedophilia who have been convicted of child sexual offences and comparison groups. The research questions were: (1) Can eye-tracking techniques detect differences in sexual preferences between individuals with paedophilia convicted of child sexual offences and comparison groups? and (2) Do individuals with paedophilia convicted of child sexual offences show a preference for child stimuli over adult stimuli in eye-tracking outcomes? In the absence of previous meta-analytic research, this study was exploratory and did not formulate *a priori* hypotheses.

## 2 Methods

The study is reported in accordance with the Preferred Reporting Items for Systematic Reviews and Meta-Analyses (PRISMA) guidelines (Page et al., 2021).

### 2.1 Inclusion criteria

The study selection was based on a search, screening, and inclusion process previously conducted for a scoping review on the use of physiological biomarkers (including eye-tracking) in forensic psychiatry (Mens et al., 2025). The inclusion criteria for the current study align with the broader set of criteria established for this scoping review. Eligible studies included those involving forensic patients diagnosed with a paedophilic disorder who have been charged with or convicted of a child sexual crime, with no restrictions on gender, age, or ethnicity for participants. The studies had to be conducted within forensic psychiatry contexts, including forensic in- and outpatient clinics, correctional facilities, and other professional environments where forensic patients are assessed. Furthermore, the studies had to report on the use of eye movements, more specifically fixation latency and viewing time, as a proxy for sexual interest, assessed with eye-tracking techniques. Included were original studies (primary research) published in scientific journals and dissertations. Systematic reviews and meta-analyses (secondary studies) were excluded but were used as sources to identify additional primary research. There were no language restrictions, and translation issues were manageable.

### 2.2 Search strategy and study selection

In the scoping review, a comprehensive search was conducted across the scientific databases MEDLINE (PubMed), PsycINFO, Embase, Social Science Citation Index (Web of Science), Scopus (for citation tracking), Cochrane Library, and Google Scholar, covering all dates up to September 1, 2023. For this review, a three-step search strategy was applied. First, a limited search of MEDLINE (PubMed) and PsycINFO was conducted to retrieve relevant publications. Text words in the titles and abstracts of these articles, as well as the index terms used to describe them, were analysed to develop various iterations of a full search strategy for MEDLINE (see Appendix I). Second, a search using all identified keywords and index terms was conducted in all databases. For Google Scholar, a modified query with fewer characters and no truncation was used (see Appendix II). Third, the reference lists of systematic reviews and meta-analyses that were excluded during full-text screening were examined to identify additional relevant studies.

All identified records were uploaded and processed in EndNote 20, with duplicates removed. The resulting dataset was then exported to the online systematic review platform Rayyan (Qatar Computing Research Institute, Doha, Qatar). Titles and abstracts were screened against the scoping review’s inclusion criteria by two reviewers. To ensure consistency, the first 50 records were jointly assessed to reach consensus on the interpretation and application of the selection criteria. Subsequently, 500 records were independently screened by both reviewers, after which the ratings were compared. Inter-rater reliability was high (Cohen’s kappa = 0.94) (McHugh, 2012). The remaining records were divided between the two reviewers and screened individually. In case of doubt, a cross-check was done. Discrepancies were resolved during biweekly consensus meetings. The full texts of the first 60 eligible citations were independently assessed in detail against the inclusion criteria by both reviewers. Again, inter-rater reliability was near perfect (Cohen’s kappa = 0.97) (McHugh, 2012). The remaining full texts were assessed by one of the two reviewers, with cross-checking and joint decision-making in case of uncertainty.

For the present study, an additional search was performed to identify any newly published studies. The final search terms used in the scoping review were modified to align with the current review’s inclusion criteria (see Appendix III). Specifically, all terms referring to physiological biomarkers other than ‘eye-tracking’ or ‘eye movement’ were removed. Additionally, we refined the search terms for psychiatric diagnoses to focus exclusively on studies involving individuals with paedophilic or paraphilic diagnoses. Using these modified search terms a new search was then conducted in the aforementioned scientific databases, covering all dates from September 1, 2023, to January 18, 2026. In addition, the studies already included in the scoping review were again screened using the narrower inclusion criteria of the current study. Additionally, the list of included studies from systematic reviews and meta-analyses was re-screened to identify additional studies involving eye-tracking in individuals diagnosed with paedophilia who have been suspected or convicted of child sexual offences.

### 2.3 Data collection and data items

Data were extracted using a data extraction tool developed by the researchers based on the JBI instrument for extracting study details and results (Peters et al., 2020). The following data were extracted for the samples of individuals diagnosed with paedophilia and suspected or convicted of child sexual crimes: the recruitment setting, number of participants (N), mean age, gender, and presence of a diagnosis of paedophilic disorder. For the control groups, information was collected on the type of comparison group, number of participants (N), mean age, and gender. Additionally, data were extracted on the instruments and stimuli used to assess sexual preference, the reported outcome(s), and the statistical analyses applied.

### 2.4 Quality assessment

Quality was assessed using the revised Quality Assessment of Diagnostic Accuracy Studies tool, Second Edition (QUADAS-2; Whiting et al., 2011), which the Cochrane Collaboration recommends for appraising prognostic and diagnostic accuracy studies (Reitsma et al., 2023). QUADAS-2 rates Risk of Bias in four domains (Patient Selection, Index Test(s), Reference Standard, and Flow and Timing), and Concerns about Applicability in three domains (Patient Selection, Index Test(s), and Reference Standard). Each domain was scored as “High,” “Low,” or “Unclear” based on specific predefined signalling questions. The majority of the studies included in the current review did not assess the diagnostic accuracy of eye-tracking methods. Instead, they examined outcome measures such as fixation latency and viewing time in case and control groups and compared these outcome variables between groups. This limits the tool’s ability to fully capture the methodological quality of the evidence base.

Therefore, in addition to the QUADAS-2, we used the Newcastle-Ottawa Scale (NOS, Wells et al., 2011), which is recommended by the Cochrane Collaboration for assessing the methodological quality of case–control studies (Reitsma et al., 2023). According to the Cochrane Handbook (Reitsma et al., 2023), it is allowed to add signalling questions within an existing domain if relevant issues are not adequately addressed by the QUADAS-2 tool. Following this guidance, and after careful consideration, we decided to integrate specific NOS items related to the selection of control groups into the Patient Selection domain of the QUADAS-2 tool. More specifically, the NOS items on Selection of Controls, and Definition of Controls and Comparability were incorporated as additional signalling questions. This modification allowed for a more nuanced assessment of both risk of bias and applicability concerns for the patient and control groups. The added questions were formulated in the same style as those in the standard QUADAS-2 tool, facilitating standardised reporting. The final version of the adapted quality assessment tool is available in Appendix V. Two independent reviewers conducted the quality assessments. Discrepancies were resolved through discussion.

### 2.5 Effect measures and synthesis methods

Three clusters of meta-analyses were conducted. In cluster 1, individuals with paedophilia convicted of child sexual offences (PCSO) were compared to healthy controls (HC) and controls without clinical signs of paedophilia. Viewing time was used as a proxy for sexual interest, differentiated by child and adult stimuli. The latter comparison group used in the meta-analyses was heterogeneous and consisted of individuals convicted of adult sexual offences (SOAA), volunteers, and individuals convicted of child sexual offences, all of whom exhibited no clinical signs of paedophilia. These control groups differed in terms of forensic status, sexual offending history, and recruitment context, and should not be considered as a single homogeneous comparison group. More specifically, the SOAA control group from Vásquez Amézquita et al. (2019) was included; participants in this group were excluded when they had any history of sexual offences against children and scored more than one on any of the seven items of the Children and Sex Cognitive Distortion Scale. Additionally, the control group reported by Kamenskov and Yakovchik (2020) included both volunteers (*N* = 21) and matched individuals who had committed sexual offences against children (*N* = 36), all without clinical signs of paedophilia. In Fromberger et al. (2012), the forensic control group consisted of inpatients with a history of sexual offences against adults but without a diagnosis of paedophilia. In cluster 2, PCSOs were compared to HC, SOAA, offenders, and controls without clinical signs of paedophilia, using fixation latency as the outcome measure. In cluster 3, differences in preference for adult versus child stimuli within the PCSO groups were examined, again using viewing time and fixation latency as outcome measures. In none of these meta-analyses were the child and adult stimuli further differentiated by gender (male, female, boy, girl) or by area of interest (e.g., head, breasts, waist, pubic region). This reflected incomplete and non-comparable data across the included studies. The number of fixations was not included as an outcome measure in the current meta-analyses because only two studies reported this variable (Ilyina et al., 2022; Vásquez Amézquita et al., 2019). Additionally, Kamenskov and Yakovchik (2020) reported first fixation duration, mean fixation duration, and the fixation activity index. As these outcome measures were not reported in any other study, they were also excluded from the meta-analyses.

Because some studies employed different measures of viewing time (e.g., total fixation time versus total duration of fixations), the pooled effect size, Hedges’ g, was used instead of the raw mean differences in all meta-analyses. Hedges’ g, recommended for small sample sizes (Durlak, 2009; Hedges, 1981), was calculated as follows: The mean of the control sample (*M*_1_) was subtracted from the mean of the PCSO sample (*M*_1_) and divided by the pooled standard deviation, *g* = (*M*_1_ − *M*_2_)/√[(*n*_1_−1)*SD_1_^2^ + (*n*_2_−1)*SD_2_^2^]/(*n*_1_ + *n*_2_−2). If a study did not report SDs, Hedges’ g was calculated using the *t*-statistic: *g* = *t* {[√(*n*_1_ + *n*_2_)]/(√*n*_1_*n*_2_)} (Rosenthal and Rosnow, 1991). For small sample sizes, *N* < 50 (where *N* = *n*_1_ + *n*_2_), g was multiplied by the correction factor J = 1–3/(4 *N*-9) to reduce upwards bias. Standard errors for the forest plots were also calculated: SE_g_ = √[(*n*_1_ + *n*_2_)/*n*_1_*n_2_ + *g*^2^/2(*n*_1_ + *n*_2_)]. A Hedges’ g of 0.2, 0.5, and 0.8 was interpreted as a small, moderate, and large effect size, respectively (Kabir et al., 2024). Effects were tested at *α* = 0.05.

Statistical heterogeneity was assessed by investigating the overlap of point estimates and their 95% confidence intervals. A random effects model was used to account for systematic variance between studies (heterogeneity) (Kabir et al., 2024). First, heterogeneity was quantified using *Q*-statistics (with significant $x^{2}$ value indicating heterogeneity) and the *I^2^* index. *I^2^* values between 0 and 40% may indicate low heterogeneity, 30–60% moderate, 50–90% substantial, and between 75 and 100% considerable heterogeneity (Dettori et al., 2021; Higgins et al., 2024). Due to the low number of studies (<10) per analysis, publication bias was not assessed. All analyses were conducted using IBM SPSS Statistics V29 (Kabir et al., 2024).

The authors take responsibility for the integrity of the data, the accuracy of the data analyses, and have made every effort to avoid inflating statistically significant results. For the current exploratory meta-analysis, no research ethics approval was required.

## 3 Results

### 3.1 Study selection

**Figure 1:**
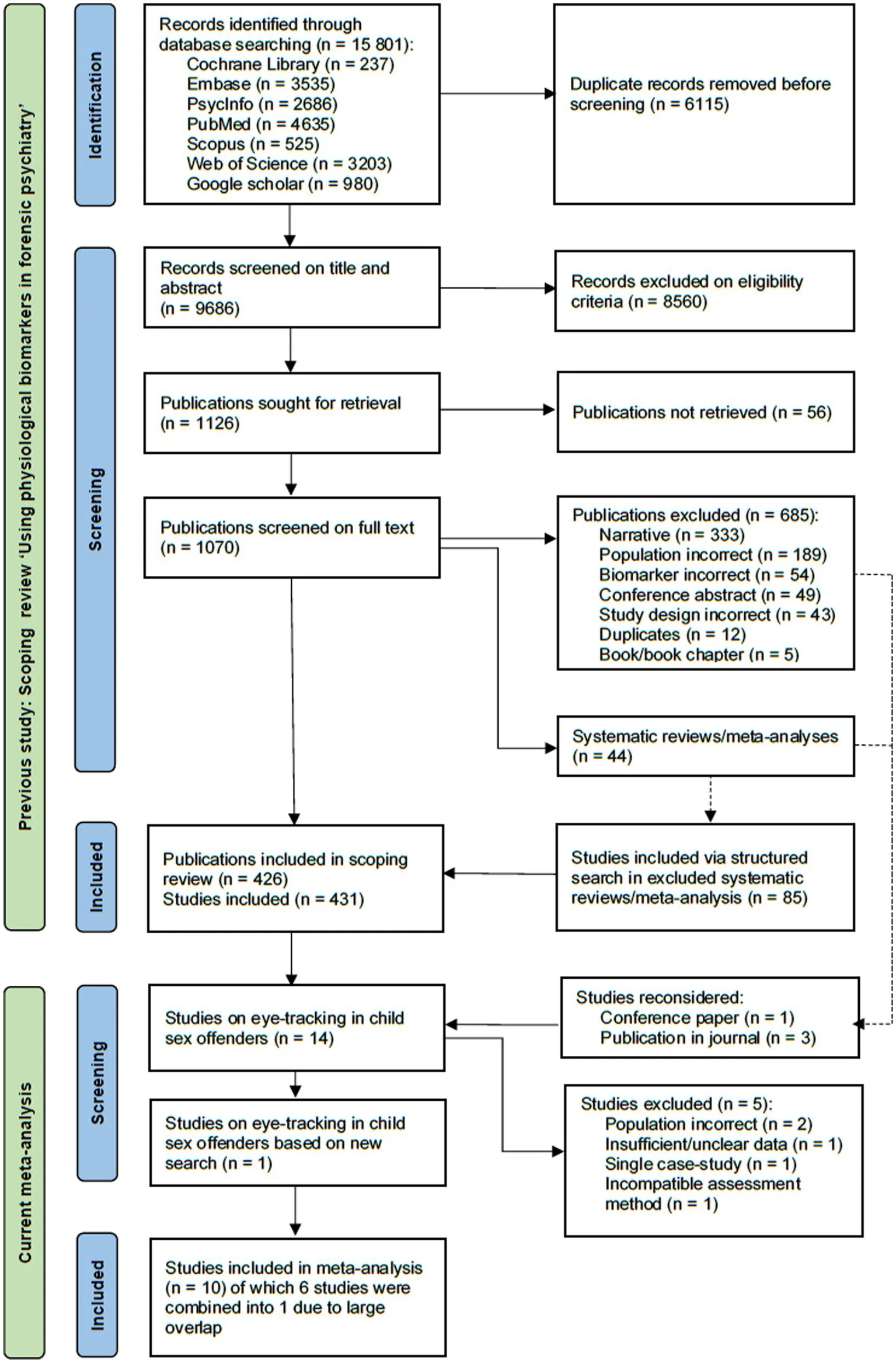
Flowchart study selection.

Figure 1 presents the study selection flowchart from the scoping review previously conducted. A total of 15,801 records were retrieved from database searches (Mens et al., 2025). After removing duplicates, 9,686 records were screened based on titles and abstracts. Subsequently, 1,070 publications were assessed for full-text eligibility. At the end of the selection process, 431 studies from 426 publications (some of which contained more than one qualifying study) were included in the scoping review. Of these, 85 studies were added via a structured reference search of excluded systematic reviews and meta-analyses.

Among all the included studies of the scoping review, nine met all the inclusion criteria. Of these studies eligible for inclusion, five were part of a large research project (Fromberger et al., 2012, 2013; Jordan et al., 2018; Jordan et al., 2016a, b). In the current exploratory meta-analysis, these articles were treated as a single study due to overlapping data, including shared patient populations, partially overlapping control groups, and similar study designs. Given the overlap in samples across studies, we adopted this conservative approach to avoid double-counting data. Data from Fromberger et al. (2012) were selected for analysis, as they provided the most comprehensive and informative results.

Furthermore, a new database search using the adjusted search terms covering studies published up to January 18, 2026, identified one additional study (Jordan et al., 2025). However, full-text examination revealed that this study was also part of the same research project as the studies identified previously. Consequently, it was considered part of the same body of work as the studies by Fromberger et al. (2012, 2013), Jordan et al. (2016a, b), and Jordan et al. (2018). After accounting for this overlap, a total of five independent studies were included in the present exploratory meta-analysis (see Figure 1).

### 3.2 Description of study characteristics

Table 1 summarises the characteristics of the included studies (highlighted in bold). It also lists all other studies associated with the same research project as reported in Fromberger et al. (2012), thereby clarifying overlaps in data and study designs. Five of these overlapping studies used the same PCSO group, while only Jordan et al. (2025) examined a slightly different patient population. More specifically, this study included 11 additional paedophilic individuals convicted of child sexual offences, increasing the PCSO group to a total of 33 participants. Across all six overlapping studies, the control groups were also highly comparable. Fromberger et al. (2012, 2013) examined the same forensic and healthy control groups. Similarly, Jordan et al. (2016a, b) used these control groups, although this study excluded more participants, resulting in reduced sample sizes. Jordan et al. (2018) merged these two control groups into a single group and additionally introduced a group consisting of outpatients with self-reported sexual interest in children. More recently, Jordan et al. (2025) expanded this outpatient group from 11 to 26 participants and doubled the number of forensic controls who had not committed child sexual offences to 14 participants. All six studies used the same eye-tracking instrument; however, the outcome variables differed. Five studies examined fixation latency, while three studies examined relative fixation time. Jordan et al. (2025) examined absolute first fixation as an outcome measure rather than fixation latency. In conclusion, comparison of these six studies, all related to the same research project, reveals extensive reuse of the same population groups, datasets, and experimental procedures. Since this raises concerns about potential data duplication and the independence of the research findings, these articles were treated as a single study, as noted in the study selection process description. This approach may have inflated the apparent consistency of findings and limited the independence of the pooled estimates.

**Table 1:** Study characteristics.

Study	Individuals who have committed sexual offences against children (all males)				Comparison groups (all males)			Assessment of sexual preference		
	Setting	*N*	Mean age	Diagnosis	Group(s)	*N*	Mean age	Instrument	Stimuli	Outcome(s)
**Fromberger et al. (****2012**, **2013)**	Forensic inpatient clinic	22	42.06	Paedophilic disorder	Forensic controls^1^ Healthy controls	8 52	36.88 25.27	SMI iView X™ RED eye-tracker	Paired adult and child pictures from NRP set^2^	Fixation latency^3^ Relative fixation time
**Ilyina et al. (** **2022)**	Correctional facility	10	NS	Paedophilic disorder	Heterosexual controls	26	NS (range 18–45)	SMI iView XTM RED eye-tracker	Alternating paired pictures of dressed adult women with either a girl or a boy	Total fixation (viewing) time Number of fixations
Jordan et al. (2018)	Forensic inpatient clinic	22	42.09	Paedophilic disorder	Outpatients with self-reported sexual interest in children Forensic controls^1^ and healthy controls combined	11 60 (8 + 52)	39.18 26.82	SMI iView XTM RED eye-tracker	Paired adult and child pictures from NRP set^2^ (initial orientation) Mental rotation stimulus (cognitive task) and sexual distractor stimuli: non-paired pictures of dressed and nude female and male adults and children from NRP set^2^	Fixation latency
Jordan et al. (2016a, 2016b)	Forensic inpatient clinic	22	42.09	Paedophilic disorder	Forensic controls^1^ Healthy controls	7 50	34.86 25.38	SMI iView X RED eye-tracker	Mental rotation stimulus (cognitive task) and sexual distractor stimuli: non-paired pictures of male adults and children from NRP set^2^	Fixation latency Relative fixation time
Jordan et al. (2025)	Forensic inpatient clinic	33	40.58	Paedophilic disorder^4^	Non-forensic persons of whom most have committed sexual offences against children^5^ Forensic persons who have not committed sexual offences against children Non-forensic persons who have not committed sexual offences against children	26 14 53	40.65 36.93 26.30	SMI iView X RED eye-tracker	Mental rotation stimulus (cognitive task) and sexual distractor stimuli: non-paired pictures female and male adults and children from NRP set^2^	Absolute first fixation Relative fixation time
**Kamenskov and Yakovchik (** **2020)**	Forensic inpatient clinic	43	37.20	Paedophilic disorder	Forensic controls and volunteers combined	57	36.90	SMI iView X RED eye-tracker	Pictures of nude female models and children of both sexes	Entry time Viewing time First fixation duration Mean fixation duration Fixation activity index
**Pezzoli et al. (** **2021)**	Outpatient sexual behaviours clinic	16	43.2	Paedophilic disorder	Healthy controls (community sample)	16	43.4	SMI RED 250 eye-tracker	Paired pictures of clothed and nude female and male children and adults from NRP^2^ and VPS sets^6^; Advertising photographs of female and male children and adults wearing swimsuits	Response (fixation) latency
**Vásquez Amézquita et al. (** **2019)**	Correctional facility	18	44.5	Paedophilic disorder presumed^5^	Sex offenders against adults Nonsexual offenders Non-offenders	16 18 19	40.25 39.22 40.42	Tobii Pro X2-60 eye-tracker integrated with Tobii Studio Pro Version 3.3.2	Paired pictures of semi-clothed (underwear or bathing suit) adult females and girls and adult males and boys retrieved from freely accessible Internet sites	Fixation latency^7^ Number of fixations^7^ Total fixation (viewing) time^7^

Included studies in the current meta-analysis are highlighted in bold. NS = Not Specified; ^1^Male forensic inpatients who sexual offended against adults but not children; ^2^Stimuli were selected from the Not-Real-People (NRP) Picture Set which contains pictures of nude and clothed male and female persons at five different stages of puberal development based on Tanner’s categorization (Pacific Psychological Assessment Corporation, 2004); ^3^Areas of interest: entire person, head, breasts, waist, pubic region; only intra-group pairwise comparisons; ^4^Of the 33 participants, 27 were diagnosed with pedophilia, representing 81.8%, ^5^Individuals being treated in the outpatient preventive project ‘PsM’ with self-reported sexual interests in children and/or sexual offences against children. ^6^Virtual People Set (VPS): idem (Dombert et al., 2013); ^5^Sexual offenders against children with a score of 3 or 4 on each of seven items selected by the authors of the Children and Sex Cognitive Distortions Scale (Waldron et al., 2006); ^7^ Areas of interest: entire body, face, chest, pelvis; inter-group comparisons. Included studies in the current meta-analysis are highlighted in bold.

Among the five studies included in the current exploratory meta-analysis, two were conducted in correctional facilities, two in a forensic inpatient setting, and one in an outpatient clinic. All participants in both the PCSO and control groups were male. The sample sizes of the PCSO groups ranged from 10 to 43 participants, with an average age of 41.74 years, noting that age data were missing in one study. All studies included participants in the PCSO group only if they had been officially diagnosed with a paedophilic disorder, except for Vásquez Amézquita et al. (2019). In this study, prisoners convicted of child sexual offences were compared with several control groups. Although the offenders had not been formally diagnosed with a paedophilic disorder, the authors assessed paedophilic interests using seven items from the Children and Sex Cognitive Distortions Scale (Waldron et al., 2006). Only participants scoring 3 or 4 (maximum score) on all seven items were included in the Sexual Offenders Against Children (SOAC) group. This stringent inclusion criterion makes the assumption that a paedophilic disorder is sufficiently plausible, justifying the study’s inclusion in the analysis.

The sample sizes of the control groups varied from 8 to 57 participants. Across all control groups, the average age was 37.48 years. Each of the included studies compared the PCSO group with one or more control groups, including incarcerated individuals who sexually offended against adults, incarcerated non-sexual offenders, forensic inpatients who sexually offended against adults, forensic inpatients with a history of sexual offending but without signs of a paraphilic diagnosis, healthy controls or individuals who had committed sexual offenses but without clinical signs of paraphilia, as well as volunteers. These control groups differed in background characteristics and should not be treated as one homogeneous group.

Many included studies used the SMI iView XTM RED eye-tracker, whereas only Vásquez Amézquita et al. (2019) employed a different eye-tracking device. Across studies, participants’ visual attention responses to adult and/or child stimuli were recorded. However, the methods used to present these stimuli varied considerably between studies. Three studies used pairs of male stimuli (pictures of men paired with pictures of boys) or pairs of female stimuli (pictures of women paired with pictures of girls). In one study, pictures of women were paired with pictures of girls or boys, whereas in another study, pictures of women, boys, and girls were presented separately. All adult and child stimuli were either fully clothed, dressed in underwear or swimsuits, or nude. Three studies examined the visual attention responses to the overall stimuli, whereas two studies examined both the overall stimuli and specific areas of interest, such as the head, breasts, and pubic area.

Outcome measures used as proxies for assessing sexual preference included fixation latency, absolute first fixation, viewing time, relative fixation time, and number of fixations. However, both the selection and definitions of these measures varied across studies. In the current exploratory meta-analysis, only fixation latency and viewing time were considered outcome measures, as they were the most informative and consistently reported variables. Fixation latency was used as an indicator of early attentional processes, although terminology differed across studies. For example, Fromberger et al. (2012) and Vásquez Amézquita et al. (2019) defined fixation latency as the time taken from stimulus onset to the first fixation within an area of interest. Kamenskov and Yakovchik (2020) used the term “entry time” instead of fixation latency to refer to the duration from the start of stimulus onset until the gaze enters the area of interest. Likewise, Pezzoli et al. (2021) labelled this measure “response latency,” defining it as the time from stimulus onset to the first fixation on the target. Ilyina et al. (2022) did not include fixation latency as an outcome measure; however, they did include the total duration of fixations (viewing time) without providing a specific definition. Similarly, Vásquez Amézquita et al. (2019) examined the total duration of fixations, defined as the average total time spent looking at an area of interest. Kamenskov and Yakovchik (2020) operationalised viewing time as the sum of the durations of all saccades and fixations within an area of interest. Across all these studies that included viewing time, it was consistently viewed as an indicator of late attentional processes. Fromberger et al. (2012) and Pezzoli et al. (2021) also included viewing time in their analyses; however, instead of measuring eye movements, they used participants’ response times as a proxy for viewing time. More specifically, Fromberger et al. (2012) measured the time from stimulus onset to the completion of a self-reported sexual arousal rating of the presented stimuli, while Pezzoli et al. (2021) asked participants to indicate the location of visual stimuli, using response time as an indirect measure of viewing time. These outcome variables were not included in the meta-analyses.

Fromberger et al. (2012) reported that fixation latency measured with eye-tracking was able to distinguish individuals diagnosed with paedophilia from those without, with high reported sensitivity and specificity. Ilyina et al. (2022) reported that participants diagnosed with paedophilia displayed a greater number of fixations on pictures of children. Additionally, the total fixation duration on these child stimuli was significantly longer in the paedophilic group than in the control group. Vásquez Amézquita et al. (2019) found that individuals convicted of child sexual offences with paedophilic interest showed more first fixations on child stimuli and longer fixation duration compared to forensic controls, although no significant differences were observed compared with the healthy control group. Kamenskov and Yakovchik (2020) reported that the duration of the first fixation was longer for stimuli that matched participants’ sexual preference. However, not all results supported this pattern. Pezzoli et al. (2021) did not observe an automatic attention bias toward child stimuli, nor were any effects of sexual preference found. Overall, the available evidence indicates that eye-tracking may provide a promising complementary approach for the indirect assessment of paedophilic sexual interest. However, the current evidence base remains limited and heterogeneous, with substantial variation in study designs, sample characteristics, stimuli, and outcome measures. Therefore, the findings should be interpreted with caution, and further well-designed studies are needed before firm conclusions can be drawn regarding the value of eye-tracking as an assessment method.

### 3.3 Quality assessment

The results from the quality assessment are presented in Table 2.

**Table 2:** Results of the adapted QUADAS-2 assessment.

Study	Risk of bias						Applicability concerns				
	Patient selection	Control selection	Index test 1	Index test 2	Reference standard	Flow and timing	Patient selection	Control selection	Index test 1	Index test 2	Reference standard
Fromberger et al. (2012)	High	High	Low	Low	Low	Low	Low	High	Low	Low	Low
Ilyina et al. (2022)	High	Unclear	NA	Low	Low	Low	Low	Unclear	NA	Low	Low
Kamenskov and Yakovchik (2020)	High	High	Low	Low	Low	Low	Low	High	Low	Low	Low
Pezzoli et al. (2021)	High	High	Low	NA	Low	Low	Low	High	Low	NA	Low
Vásquez Amézquita et al. (2019)	High	High	Low	Low	Low	Low	Low	High	Low	Low	Low

NA = not applicable. Index test 1 = fixation/response latency and Index test 2 = viewing time.

Four out of five studies demonstrated a high risk of bias in the methodological quality of both patient and control selection (Fromberger et al., 2012; Kamenskov and Yakovchik, 2020; Pezzoli et al., 2021; Vásquez Amézquita et al., 2019) in the remaining study, the risk of bias related to control selection was unclear (Ilyina et al., 2022). All studies included non-consecutive samples and employed a case–control design. For patient selection, two studies made inappropriate exclusions without transparent justification (Fromberger et al., 2012; Pezzoli et al., 2021); another study listed exclusion criteria but failed to report how many participants were excluded (Ilyina et al., 2022); only one study specified both the number and the reasons for exclusion (Vásquez Amézquita et al., 2019). One study did not provide any information regarding excluded participants (Kamenskov and Yakovchik, 2020). For control selection, one study avoided inappropriate exclusions (Vásquez Amézquita et al., 2019), whereas the other studies did not describe their procedures in sufficient detail. In addition, none of the reviewed studies recruited control participants who met the NOS definition of community controls (i.e., individuals drawn from the same population as cases and who would have been cases had they developed the outcome). Nonetheless, each study documented the controls’ outcome history. Regarding comparability, one study statistically controlled for age, IQ, and hospitalization (Fromberger et al., 2012); another study controlled for age and matched groups based on years of education, handedness, and global attentional capacity (Pezzoli et al., 2021); one study reported that the three offender groups were similar in age and education (Vásquez Amézquita et al., 2019); the remaining two provided no information on comparability (Ilyina et al., 2022; Kamenskov and Yakovchik, 2020). For the Index Test and Reference Standard domains, all five studies were rated as low risk of bias. Each study employed objective eye-tracking measures, such as viewing time and/or fixation latency, to differentiate cases from controls. Because the interpretation of test results was automatically generated, the signalling question regarding blinding to the reference standard was marked as “Not Applicable.” Likewise, no study used a prespecified diagnostic threshold, warranting a second “Not Applicable” designation. In the Flow and Timing domain, all studies described a clear and uninterrupted testing sequence: all participants received the same reference standard immediately after or concurrently with the index test, and no attrition was reported. Consequently, the risk of bias in this domain was judged to be low across all studies. Taken together, these limitations suggest that selection bias is a serious concern and may limit the external validity of the pooled estimates reported in the present synthesis of findings overall.

All patients included in the five studies were consistent with the participants described in the review question, raising no concerns about the applicability of patient selection. In one study, the diagnostic method was not explicitly described. However, the researchers indicated that participants had been diagnosed with paedophilia, were receiving treatment at an outpatient sexual behaviour clinic, and were currently on bail, probation, or parole for sexual offences against children under the age of 16 (Pezzoli et al., 2021). Furthermore, these individuals self-reported a sexual interest in children. Another study did not use a formally validated diagnostic procedure but included individuals who had been convicted or sentenced for offences against minors and scored high on cognitive distortion scales related to paedophilia (Vásquez Amézquita et al., 2019). Based on this information, it is reasonable to assume that the included patients aligned with the review question. In terms of controls, one study lacked sufficient information about the control group, making the applicability concerns unclear (Ilyina et al., 2022). In the remaining studies, limited information on the selection procedures made it difficult to determine whether healthy participants were representative of the general population, resulting in high concerns regarding the applicability of the control group selection. No concerns were identified regarding the applicability of the index tests or the reference standard in any of the five studies.

An overview of the raw data used to calculate effect sizes and standard errors for all five included studies is available in Appendix VI.

### 3.4 Meta-analyses

#### 3.4.1 Cluster 1: viewing time of PCSO vs. HC and controls without clinical signs of paedophilia

Two included studies examined differences in viewing time between PCSO and HC using adult stimuli (Ilyina et al., 2022; Vásquez Amézquita et al., 2019). The pooled effect size was not statistically significant (Hedges’ *g* = −0.15, 95% CI: −0.63 to 0.34, *p* = 0.55) (see forest plot Supplementary Figure 2). Visual inspection shows that the point estimates and confidence intervals across the studies differ somewhat, but not substantially. Also, the *Q*-statistic was not statistically significant ( $x^{2}=0.24$ , *p* = 0.62) and *I^2^* < 0.01 suggests little evidence of heterogeneity between studies. The same studies also examined differences in viewing time between PCSO and HC using child stimuli. Although both Ilyina et al. (2022) and Vásquez Amézquita et al. (2019) reported that PCSO fixated longer on these stimuli than control groups, overall, the pooled effect size was not statistically significant (Hedges’ *g* = 0.40, 95% CI: −0.09 to 0.89, *p* = 0.11) (see forest plot Supplementary Figure 3). In this meta-analysis, the heterogeneity between studies appeared to be limited ( $x^{2}=0.12$ , *p* = 0.73, *I^2^* < 0.01).

In two studies (Kamenskov and Yakovchik, 2020; Vásquez Amézquita et al., 2019), viewing times for PCSO and a mixed control group consisting of individuals who did not exhibit clinical signs of pedophilia were compared using adult and child stimuli. Both meta-analyses produced statistically insignificant pooled effect sizes (adult stimuli: Hedges’ *g* = 2.44, 95% CI: −1.5 to 6.38, *p* = 0.22; child stimuli: Hedges’ *g* = −1.47, 95% CI: −6.00 to 3.07, *p* = 0.53). Additionally, in both meta-analyses, there were clear indications of considerable heterogeneity among studies (adult stimuli: $x^{2}=52.46$ , *p* < 0.01, *I^2^* = 0.98; child stimuli: $x^{2}=67.70$ , *p* < 0.01, *I^2^* = 0.99) (see forest plots Supplementary Figures 4, 5).

#### 3.4.2 Cluster 2: fixation latency of PCSO vs. HC, SOAA, offenders, and controls without clinical signs of pedophilia

Two studies (Pezzoli et al., 2021; Vásquez Amézquita et al., 2019) that examined differences in fixation latency on adult stimuli between PCSO and HC provided opposite trends, both not statistically significant, which also applied to the pooled effect size (Hedges’ *g* = −0.05, 95% CI: −1.17 to 1.07, *p* = 0.93). Additionally, there were clear indications of considerable heterogeneity among these studies ( $x^{2}=5.03$ , *p* = 0.02, *I^2^* = 0.80) (see forest plot Supplementary Figure 6). Moreover, a third study, which did not provide raw fixation latency data for adult stimuli but did present visualisations, found no significant differences in fixation latencies among samples of PCSO, HC, and forensic controls (Fromberger et al., 2012). Concerning fixation latency time for child stimuli, the pooled effect size of differences between PCSO and HC was not statistically significant (Hedges’ *g* = −0.35, 95% CI: −0.75 to 0.05, *p* = 0.09), with limited heterogeneity between studies ( $x^{2}=2.51$ , *p* = 0.29, *I^2^* = 0.23) (Fromberger et al., 2012; Pezzoli et al., 2021; Vásquez Amézquita et al., 2019) (see forest plot Supplementary Figure 7).

However, significant large, pooled effect sizes were found for the same comparison between PCSO and SOAA and Offender control samples, indicating that PCSO fixate faster on child stimuli than both control groups (SOAA: Hedges’ *g* = −0.88, 95% CI: −1.55 to −0.21, *p* = 0.01; Offenders: Hedges’ *g* = −0.87, 95% CI: −1.50 to −0.24, *p* = 0.01) (see forest plots Supplementary Figures 8, 9) (Fromberger et al., 2012; Vásquez Amézquita et al., 2019). The aforementioned analyses showed limited indications for heterogeneity, and if any, it might not be important (SOAA: $x^{2}=1.49$ , *p* = 0.22, *I^2^* = 0.33; Offenders: $x^{2}=1.54$ , *p* = 0.21, *I^2^* = 0.35). No results could be calculated for adult stimuli due to unreported data.

The same comparison was made between PCSO and a mixed control group consisting of individuals who did not exhibit clinical signs of pedophilia using adult and child stimuli (adult stimuli: Kamenskov and Yakovchik, 2020; Vásquez Amézquita et al., 2019; child stimuli: Fromberger et al., 2012; Kamenskov and Yakovchik, 2020; Vásquez Amézquita et al., 2019). The pooled effect size regarding adult stimuli was not statistically significant (Hedges’ *g* = −2.07, 95% CI: −5.43 to 1.28, *p* = 0.23) (see forest plot Supplementary Figure 10). Furthermore, this analysis showed indications of considerable heterogeneity between studies ( $x^{2}=51.32$ , *p* = <0.01, *I^2^* = 0.98). However, when comparing PCSO and the mixed control group using child stimuli, a significantly large, pooled effect size was found (Hedges’ *g* = −1.01, 95% CI: −1.43 to −0.6, *p* < 0.01) (see forest plot Supplementary Figure 11). These results indicate that PCSOs fixated faster on child stimuli compared to individuals without clinical signs of paedophilia. This analysis showed limited indications for heterogeneity ( $x^{2}=2.71$ , *p* = 0.26, *I^2^* = 0.26).

#### 3.4.3 Cluster 3: differences in preference for adult and child stimuli in PCSO

The pooled effect size for differences in viewing time using adult and child stimuli in PCSO was statistically significant (Hedges’ *g* = 1.69, 95% CI: 0.58 to 2.81, *p* < 0.01) (Ilyina et al., 2022; Kamenskov and Yakovchik, 2020; Vásquez Amézquita et al., 2019). These results indicate that PCSO fixated longer on child stimuli compared to adult stimuli (see forest plot Supplementary Figure 12). Finally, results from four studies showed no trend for PCSO to fixate more quickly on child than on adult stimuli (Fromberger et al., 2012; Kamenskov and Yakovchik, 2020; Pezzoli et al., 2021; Vásquez Amézquita et al., 2019). The pooled effect size was not statistically significant (Hedges’ *g* = −0.58, 95% CI: −1.89 to 0.74, *p* = 0.39) (see forest plot Supplementary Figure 13). Furthermore, there are indications of considerable heterogeneity between the studies in both analyses (viewing time: $x^{2}=15.99$ , *p* = <0.01, *I^2^* = 0.85; fixation latency: $x^{2}=58.37$ , *p* < 0.01, *I^2^* = 0.94).

## 4 Discussion

This exploratory meta-analysis aimed to evaluate whether eye-tracking measurements are associated with paedophilic sexual preferences by examining group-level differences between individuals diagnosed with paedophilia who have been convicted of child sexual offences (PSCO) and various comparison groups. Data on viewing time and fixation latency for adult and child stimuli were used as input for the meta-analyses. Results suggested that PCSOs displayed distinct attentional patterns toward child-related stimuli in early fixation measures when compared to individuals convicted of adult sexual offences (SOOA), offenders and individuals without clinical signs of paedophilia. This significant distinction was not found when comparing PCSOs to healthy community controls (HC), indicating moderate specificity of eye-tracking metrics in this context. No significant differences in viewing time were observed between PCSOs and comparison groups. However, the meta-analyses addressing the second research question demonstrated that participants in the PCSO group spent more time viewing child stimuli than adult stimuli. This apparent discrepancy may be explained by the fact that viewing time and fixation latency capture different aspects of visual attention (Vásquez-Amézquita et al., 2023). Specifically, fixation latency is thought to reflect early attentional orienting, whereas viewing time reflects sustained attention and the relative allocation of attention to the presented stimuli.

A systematic literature search covering all publications up to 18 January 2026, followed by screening against predefined inclusion criteria, identified only ten primary research publications investigating eye-tracking in individuals with paedophilia who had been convicted of child sexual offences. Of these, six originated from the same research project and included substantially overlapping data and study designs. Overall, these findings indicate that research on eye-tracking in individuals with paedophilia who have been convicted of child sexual offences is still limited. This was already demonstrated in the systematic reviews by Godet and Niveau (2021) and Schippers et al. (2023), which included only six and four studies, respectively, on eye-tracking in PCSO populations. Collectively, findings of the current exploratory meta-analysis suggest that little new research has been conducted on this specific topic in recent years.

The first research question focused on the ability of eye-tracking measures to differentiate attentional responses associated with sexual preferences between PCSOs and various comparison groups. Results showed that early fixation measures demonstrated greater discriminative value than viewing time for distinguishing PCSOs from comparison groups. More specifically, no evidence was found that PCSOs fixate longer on child stimuli and shorter on adult stimuli compared to various control groups. However, the evidence of differences in fixation latency between PCSOs and different control groups was stronger. PCSOs fixated significantly faster on child stimuli compared to SOAAs, offenders, and individuals without clinical signs of paedophilia (significantly large effect sizes). This difference was not significant between PCSOs and HCs. Furthermore, there appeared to be insufficient evidence at the meta-level of differences in fixation speed between the PCSO and control groups on adult stimuli. Overall, this meta-analysis suggests that fixation latency may be a valuable indirect indicator of paedophilic sexual interest when differentiating PCSOs from offender comparison groups. However, the absence of significant differences between PCSOs and healthy community controls indicates that its discriminative value is limited and requires further investigation.

One possible explanation for the weaker effects in comparisons between PCSOs and HCs may lie in the heterogeneity of the HC samples, the potential lack of sexual arousal during stimulus presentation (all stimuli were 2D pictures), or ceiling effects in attention towards adult stimuli. Furthermore, differences in stimulus characteristics or settings (lab versus forensic environments) may have contributed to the attenuated findings. Contrasting these findings with relevant literature is only possible to a very limited extent. In fact, the only available material is the study by Schippers et al. (2023), which, however, differs substantially in scope and design from the present one (eye-tracking is only a modest and non-specific part of the study). Be that as it may, Schippers et al. (2023) report that PCSOs showed patterns consistent with greater interest in child-related stimuli and reduced interest in adult stimuli compared with some comparison groups, in particular, community samples.

Regarding the second research question, whether the results of the eye-tracking measurements in PCSOs are indicative of a preference for child stimuli over adult stimuli, the meta-analysis found evidence of differences in viewing time between child and adult stimuli within the PCSO group. No significant results were found for fixation latency. PCSOs spent more time looking at child stimuli than adult stimuli, but their fixation speed on child stimuli was not faster than on adult stimuli. Regarding this conclusion, it is important to acknowledge that the evidence is limited and depends on a single eye-tracking metric. Furthermore, direct comparison with previous research is difficult because comparable eye-tracking meta-analyses are lacking. Nevertheless, Schippers et al. (2023) concluded that PCSOs did not show a clear sexual preference for either children or adults.

A strength of the current study is that it is the first systematically conducted exploratory meta-analysis specifically focusing on eye-tracking in individuals with paedophilia who have been convicted of child sexual offences. Additionally, a quality assessment of all five included studies was performed using an adapted version of the QUADAS-2 tool, tailored to the study purposes and designs of all the included studies. However, these results also highlighted several limitations regarding the study quality of the included studies. Importantly, the high risk of bias in patient and control selection limits the strength of the conclusions from this exploratory meta-analysis. The frequent use of case–control designs, non-random sampling, and the lack of information on exclusion criteria raise concerns about selection bias and generalizability. Additionally, there were significant concerns about the applicability of the control groups to the review question. Two studies did not provide sufficient information on the characteristics and selection of the controls, while the representativeness of the controls raised concerns in the other three studies. For instance, one study reported that 16 controls were recruited through online advertising. However, it did not specify which online platforms were used or clarify whether only 16 individuals participated or if they were selected from a larger pool of participants. Furthermore, the heterogeneity of the control groups, including forensic and community samples as well as sexual and non-sexual offenders, may have influenced baseline levels of attentional responding and contributed to variability in the findings. The control groups differed in terms of forensic status, sexual offending history, and recruitment context. Another limitation is the small number of included studies and the relatively limited cumulative numbers of PCSOs and control participants. Given the limited number of studies (n = 5), a reliable assessment of publication bias was not feasible. Another limitation concerns the diagnostic procedures used in the included studies. Although paedophilic disorder is ideally established using a comprehensive, multimethod assessment (Seto, 2008), diagnostic procedures varied across studies and were not always reported in sufficient detail, potentially reducing the comparability of the included samples. Moreover, as mentioned earlier, participants in Vásquez Amézquita et al. (2019) who had been convicted of child sexual offences had not received a formal diagnosis of a paedophilic disorder. Instead, paedophilic interest was assessed using seven items from the Children and Sex Cognitive Distortions Scale (Waldron et al., 2006). Only participants scoring 3 or 4 (the maximum score) on all seven items were included in the Sexual Offenders Against Children (SOAC) group. Although this criterion was considered sufficiently specific to identify individuals with elevated paedophilic interests for inclusion in the present review, the absence of a formal diagnosis remains an important limitation that should be considered when interpreting the results. In addition, variability in visual stimuli across studies, such as differences in the level of explicitness, gender of depicted individuals, study design, and presentation format, may have influenced attentional responses and contributed to heterogeneity in the findings. These potential sources of variation were not systematically analysed in this review (Higgins et al., 2024). Furthermore, the meta-analysis only included data on full-body stimuli and could not differentiate between subareas of interest, such as the face, breasts or genitals, even though more specific sexual preferences may influence the results. Another limitation is the absence of baseline measures of visual attention, which were not addressed in any of the included studies. Without controlling for baseline visual attention, it cannot be ruled out that shorter fixation latencies, for example, partly reflect individual differences in general attentional speed rather than stimulus-specific attentional biases related to sexual preferences. Furthermore, eye-tracking measures assess visual attention rather than sexual preference directly (Vásquez-Amézquita et al., 2023). Although attentional allocation may reflect underlying sexual interests, it can also be influenced by non-sexual factors, including novelty, emotional salience, or general attentional processes. Therefore, findings from eye-tracking studies should be interpreted with caution as indirect indicators of sexual interest and considered alongside other assessment methods, consistent with the multimethod assessment approach proposed by Seto (2008). Lastly, only one study included a comparison group of individuals who had sexually offended against children but had not been diagnosed with a paedophilic disorder. However, this group was supplemented with volunteers, resulting in a heterogeneous comparison group. As a result, it remains unclear whether eye-tracking assessments can differentiate between individuals who have sexually offended against children with and without paedophilic sexual interests, potentially supporting the theoretical literature review by Vásquez-Amézquita et al. (2023). Among all the included studies, ten focused specifically on eye-tracking in individuals who have sexually offended against children and met all the inclusion criteria. Of the studies eligible for inclusion, six were part of a large research project (Fromberger et al., 2012, 2013; Jordan et al., 2018; Jordan et al., 2016a, 2016b; Jordan et al., 2025). In the current meta-analysis, these articles were treated as a single study due to overlapping data, including shared patient populations, partially overlapping control groups, and similar study designs. Given the overlap in samples across studies, we adopted this conservative approach to avoid double-counting data. However, this approach may have resulted in loss of information, reduced statistical power, and obscured within-study variability. Approaches such as multilevel meta-analysis or robust variance estimation could allow the inclusion of multiple effect sizes per study, but were not feasible given the limited number of available studies (Cheung, 2019). In conclusion, although the results of this meta-analysis should be interpreted with caution due to a limited number of included studies and methodological differences between studies, there is some evidence that eye-tracking techniques may provide complementary information for the assessment of paedophilic sexual interest. However, more research is needed to determine the value of eye-tracking for clinical practice, which should consider not only etiological and diagnostic applications but also (effect) monitoring, assessment of recidivism risk, and even the enrichment of treatment interventions for PCSO (e.g., biofeedback). First, eye-tracking techniques may provide complementary behavioural information within a multimethod assessment framework by offering an indirect measure of attentional responses that is less dependent on self-report or clinical disclosure. This is particularly valuable in forensic populations, where individuals may minimise or deny paedophilic sexual interests (Vásquez-Amézquita et al., 2023). Second, if supported by further validation studies, eye-tracking could contribute to treatment monitoring by objectively assessing changes in attentional responses throughout cognitive-behavioural interventions. Third, eye-tracking may ultimately support risk evaluation by complementing, rather than replacing, existing assessment methods. However, given the limited evidence and methodological heterogeneity identified in the present review, future research should further investigate the psychometric properties and potential clinical applicability of eye-tracking measures in forensic populations.

## Data Availability

The original contributions presented in the study are included in the article/Supplementary material, further inquiries can be directed to the corresponding author.

## References

[ref1] Ali S. Pasha S. Cox A. Youssef E. (2024). Examining the short and long-term impacts of child sexual abuse: a review study. SN Soc. Sci. 4. doi: 10.1007/s43545-024-00852-6

[ref2] American Psychiatric Association (2022). Diagnostic and Statistical Manual of mental Disorders. 5th ed., text rev. Edn. doi: 10.1176/appi.books.9780890425787

[ref3] Bickle A. Cameron C. Hassan T. Safdar H. Khalifa N. (2021). International overview of phallometric testing for sexual offending behaviour and sexual risk. BJPsych International 18:E11. doi: 10.1192/bji.2021.17, 34747938 PMC8554943

[ref4] Buschman J. Bogaerts S. Foulger S. Wilcox D. Sosnowski D. Cushman B. (2010). Sexual history disclosure polygraph examinations with cybercrime offences. A first Dutch explorative study. Int. J. Offender Ther. Comp. Criminol. 54, 395–411. doi: 10.1177/0306624X09334942, 19389838

[ref5] Cheung M. W. L. (2019). A guide to conducting a meta-analysis with non-independent effect sizes. Neuropsychol. Rev. 29, 387–396. doi: 10.1007/s11065-019-09415-6, 31446547 PMC6892772

[ref6] Coric V. Feuerstein S. Fortunati F. Southwick S. Temporini H. Morgan C. A. (2005). Assessing sex offenders. Psychiatry (Edgmont (Pa.: Township)) 2, 26–29. PMC2993520 21120092

[ref7] Dettori J. R. Norvell D. C. Chapman J. R. (2021). Seeing the forest by looking at the trees: how to interpret a meta-analysis forest plot. Global Spine Journal 11, 614–616. doi: 10.1177/21925682211003889, 33939533 PMC8119923

[ref8] Dombert B. Mokros A. Brückner E. Schlegl V. Antfolk J. Bäckström A. . (2013). The virtual people set: developing computer-generated stimuli for the assessment of pedophilic sexual interest. Sex. Abus. 25, 557–582. doi: 10.1177/1079063212469062, 23296092

[ref9] Durlak J. A. (2009). How to select, calculate, and interpret effect sizes. J. Pediatr. Psychol. 34, 917–928. doi: 10.1093/jpepsy/jsp004, 19223279

[ref10] Fang X. Ren J. Kang J. Ligiéro D. Fry D. Annor F. B. . (2026). A systematic review of the global and regional estimates of the prevalence of sexual violence against children. Nat. Hum. Behav. 10, 1130–1142. doi: 10.1038/s41562-026-02436-1 PMC13316746 41927913

[ref11] Fromberger P. Jordan K. Steinkrauss H. von Herder J. Stolpmann G. Kröner-Herwig B. . (2013). Eye movements in pedophiles: automatic and controlled attentional processes while viewing prepubescent stimuli. J. Abnorm. Psychol. 122, 587–599. doi: 10.1037/a0030659, 23206281

[ref12] Fromberger P. Jordan K. Steinkrauss H. von Herder J. Witzel J. Stolpmann G. . (2012). Diagnostic accuracy of eye movements in assessing pedophilia. J. Sex. Med. 9, 1868–1882. doi: 10.1111/j.1743-6109.2012.02754.x, 22548761

[ref13] Gerwinn H. Weiß S. Tenbergen G. Amelung T. Födisch C. Pohl A. . (2018). Clinical characteristics associated with paedophilia and child sex offending: differentiating sexual preference from offence status. Eur. Psychiatry 51, 74–85. doi: 10.1016/j.eurpsy.2018.02.002, 29625377

[ref14] Godet T. Niveau G. (2021). Eye tracking and child sexual offenders: a systematic review. Forensic Sci. Res. 6, 133–140. doi: 10.1080/20961790.2021.1940737, 34377570 PMC8330767

[ref15] Gouveia C. Sousa M. Cunha O. Seto M. de Castro-Rodrigues A. Gonçalves R. A. (2024). Validation of the revised screening scale for pedophilic interests (SSPI-2) in Portugal. Sex. Abus. 36, 848–869. doi: 10.1177/10790632241268502, 39080999 PMC11411846

[ref16] Graham M. Friesen P. (2019). More harm than good: neurotechnological thought apprehension in forensic psychiatry. AJOB Neurosci. 10, 17–19. doi: 10.1080/21507740.2019.1595787, 31070557

[ref17] Hanson R. K. Bussière M. T. (1998). Predicting relapse: a meta-analysis of sexual offender recidivism studies. J. Consult. Clin. Psychol. 66, 348–362. doi: 10.1037/0022-006X.66.2.348, 9583338

[ref18] Hanson R. K. Morton-Bourgon K. E. (2005). The characteristics of persistent sexual offenders: a meta-analysis of recidivism studies. J. Consult. Clin. Psychol. 73, 1154–1163. doi: 10.1037/0022-006X.73.6.1154, 16392988

[ref19] Hedges L. V. (1981). Distribution theory for glass’s estimator of effect size and related estimators. J. Educ. Stat. 6, 107–128. doi: 10.3102/10769986006002107

[ref20] Higgins J. P. T. Thomas J. Chandler J. Cumpston M. Li T. Page M. J. . (2024). Cochrane Handbook for Systematic Reviews of Interventions version 6.5 (updated August). Cochrane. Available online at: https://www.cochrane.org/handbook

[ref21] Ilyina V. Kamenskov M. Dvoryanchikov N. (2022). Methodological features of pedophilic disorder assessment using an eyetracker. Psychol. Law 12, 180–196. doi: 10.17759/psylaw.2022120315

[ref22] Jordan K. Müller I. Fromberger P. Dobrunz U. Franz U. Müller J. L. (2025). Similar age preference but different attentional control in mandatory hospitalized individuals who have committed sexual offenses against children and non-hospitalized individuals with self-reported sexual interest in children. Sexual Abuse. 37, 571–608. doi: 10.1177/10790632241297271, 39508505 PMC12179397

[ref23] Jordan K. Fromberger P. Müller J. (2015). Could we measure sexual interest using functional imaging? Sex. Offender Treat. 10, 1–29.

[ref24] Jordan K. Fromberger P. Müller I. Wernicke M. Stolpmann G. Müller J. L. (2018). Sexual interest and sexual self-control in men with self-reported sexual interest in children: a first eye tracking study. J. Psychiatr. Res. 96, 138–144. doi: 10.1016/j.jpsychires.2017.10.004, 29049970

[ref25] Jordan K. Fromberger P. von Herder J. Steinkrauss H. Nemetschek R. Witzel J. . (2016a). Can we measure sexual interest in pedophiles using a sexual distractor task? J. Forensic Psychol. 1:109. doi: 10.4172/2475-319X.1000109 PMC5133255 27994559

[ref26] Jordan K. Fromberger P. von Herder J. Steinkrauss H. Nemetschek R. Witzel J. . (2016b). Impaired attentional control in pedophiles in a sexual distractor task. Front. Psych. 7:193. doi: 10.3389/fpsyt.2016.00193, 27994559 PMC5133255

[ref27] Jordan K. Wild T. Fromberger P. Müller I. Müller J. L. (2020). Are there any biomarkers for pedophilia and sexual child abuse? A review. Front. Psych. 10:940. doi: 10.3389/fpsyt.2019.00940, 32038314 PMC6985439

[ref28] Kabir R. Syed H. Z. Hayhoe R. Parsa A. D. Sivasubramanian M. Mohammadnezhad M. . (2024). Meta-analysis using SPSS: a simple guide for clinicians, public health, and allied health specialists. The Evidence 2. doi: 10.61505/evidence.2024.2.1.25

[ref29] Kamenskov M. Yakovchik A. Y. (2020). Prospects for the use of a binocular eye tracking system for the diagnosis of pedophilic disorder. S. S. Korsakov J. Neurol. Psychiatry 120, 72–79. doi: 10.17116/jnevro202012009172, 33081450

[ref30] Krajbich I. Armel C. Rangel A. (2010). Visual fixations and comparison of value in simple choice. Nat. Neurosci. 13, 1292–1298. doi: 10.1038/nn.2635, 20835253

[ref31] McHugh M. L. (2012). Interrater reliability: the kappa statistic. Biochem. Med. 22, 276–282. doi: 10.11613/BM.2012.031, 23092060 PMC3900052

[ref32] Mens J. Masthoff E. Bogaerts S. Heus P. (2025). Using physiological biomarkers in forensic psychiatry: a scoping review. Front. Psych. 16:1580615. doi: 10.3389/fpsyt.2025.1580615, 40365003 PMC12069285

[ref33] Nichols H. Molinder I. (2000). Multiphasic Sex Inventory II. Fircrest, WA: Nichols & Molinder Assessments.

[ref34] Nummenmaa L. Hyönä J. Calvo M. G. (2006). Eye movement assessment of selective attentional capture by emotional pictures. Emotion 6, 257–268. doi: 10.1037/1528-3542.6.2.257, 16768558

[ref35] Pacific Psychological Assessment Corporation (2004). The Not Real People (NRP) visual stimulus set. Victoria, BC: Pacific Psychological Assessment Corporation.

[ref36] Page M. J. McKenzie J. E. Bossuyt P. M. Boutron I. Hoffmann T. C. Mulrow C. D. . (2021). The PRISMA 2020 statement: an updated guideline for reporting systematic reviews. BMJ (Clinical research ed.) 372:n71. doi: 10.1136/bmj.n71 PMC8005924 33782057

[ref37] Papalia N. Mann E. Ogloff J. R. P. (2020). Child sexual abuse and risk of revictimization: impact of child demographics, sexual abuse characteristics, and psychiatric disorders. Child Maltreat. 26, 74–86. doi: 10.1177/1077559520932665, 32573259

[ref38] Peters M. D. J. Godfrey C. McInerney P. Munn Z. Tricco A. C. Khalil H. (2020). “*Scoping reviews*,” in JBI Manual for Evidence Synthesis, eds. Aromataris E. Lockwood C. Porritt K. Pilla B. Jordan Z. (JBI). doi: 10.46658/JBIMES-24-09

[ref39] Pezzoli P. Ziogas A. Seto M. C. Jaworska N. Mokros A. Fedoroff P. . (2021). The effects of acute transcranial direct current stimulation on attentional bias in pedophilic disorder: a preregistered pilot study. Neuromodulation 24, 879–889. doi: 10.1111/ner.13285, 33006171

[ref40] Ponseti J. Granert O. Van Eimeren T. Jansen O. Wolff S. Beier K. . (2016). Assessing paedophilia based on the haemodynamic brain response to face images. World J. Biol. Psychiatry 17, 39–46. doi: 10.3109/15622975.2015.1083612, 26452682

[ref41] Reitsma J. B. Rutjes A. Whiting P. Yang B. Leeflang M. M. Bossuyt P. M. . (2023). “Assessing risk of bias and applicability,” in Cochrane Handbook for systematic Reviews of Diagnostic test Accuracy, eds. Deeks J. J. Bossuyt P. M. Leeflang M. M. Takwoingi Y.. Version 2.0 ed (London, United Kingdom: The Cochrane Collaboration), 169–201.

[ref42] Rosenthal R. Rosnow R. L. (1991). Essentials of Behavioral Research: Methods and data Analysis. 2nd Edn. New York: McGraw Hill.

[ref43] Sandland R. Balmer A. (2012). Making monsters: the polygraph, the plethysmograph, and other practices for the performance of abnormal sexuality. J Law Soc 39, 593–615. doi: 10.1111/j.1467-6478.2012.00601.x

[ref44] Schippers E. E. Smid W. J. Hoogsteder L. M. Planting C. H. M. de Vogel V. (2023). Pedophilia is associated with lower sexual interest in adults: Meta-analyses and a systematic review with men who had sexually offended against children. Aggress. Violent Behav. 69, 101813–101821. doi: 10.1016/j.avb.2022.101813, 42574925

[ref45] Schmidt A. F. Mokros A. Banse R. (2013). Is pedophilic sexual preference continuous? A taxometric analysis based on direct and indirect measures. Psychol. Assess. 25, 1146–1153. doi: 10.1037/a0033326, 23815115

[ref46] Seto M. C. (2008). Pedophilia and sexual Offending against children: Theory, Assessment, and Intervention. American Psychological Association. doi: 10.1037/11639-000

[ref47] Seto M. C. (2009). Pedophilia. Annu. Rev. Clin. Psychol. 5, 391–407. doi: 10.1146/annurev.clinpsy.032408.153618, 19327034

[ref48] Seto M. C. (2018). Pedophilia and sexual Offending against children: Theory, Assessment, and Intervention. 2nd Edn American Psychological Association. doi: 10.1037/0000107-000

[ref49] Seto M. C. (2019). The motivation-facilitation model of sexual offending. Sex. Abus. 31, 3–24. doi: 10.1177/1079063217720919, 28715948

[ref50] Seto M. C. Eke A. W. (2017). Correlates of admitted sexual interest in children among individuals convicted of child pornography offenses. Law Hum. Behav. 41, 305–313. doi: 10.1037/lhb0000240, 28383984

[ref51] Seto M. C. Sandler J. C. Freeman N. J. (2015). The revised screening scale for pedophilic interests: predictive and concurrent validity. Sex. Abus. 29, 636–657. doi: 10.1177/1079063215618375, 26680250

[ref52] Spiering M. Everaerd W. (2007). “Sexual unconscious,” in The Psychophysiology of sex, ed. Janssen E. (Indiana University Press), 166–183.

[ref53] Tan L. Grace R. C. (2008). Social desirability and sexual offenders: a review. Sex. Abus. 20, 61–87. doi: 10.1177/1079063208314820, 18420557

[ref54] Vásquez Amézquita M. Leongoméz J. D. Seto M. C. Salvador A. (2019). Differences in visual attention patterns to sexually mature and immature stimuli between heterosexual sexual offenders, nonsexual offenders, and nonoffending men. J. Sex Res. 56, 213–228. doi: 10.1080/00224499.2018.1511965, 30198780

[ref55] Vásquez-Amézquita M. Leongómez J. D. Salvador A. Seto M. C. (2023). What can the eyes tell us about atypical sexual preferences as a function of sex and age? Linking eye movements with child-related chronophilias. Forensic Sciences Research 8, 5–15. doi: 10.1093/fsr/owad009, 37712065 PMC10498142

[ref56] Vásquez-Amézquita M. Leongómez J. D. Seto M. C. Bonilla F. M. Rodríguez-Padilla A. Salvador A. (2018). No relation between digit ratio (2D:4D) and visual attention patterns to sexually preferred and non-preferred stimuli. Personal. Individ. Differ. 120, 151–158. doi: 10.1016/j.paid.2017.08.022

[ref57] Waldron B. O’Reilly G. Randall P. Shevlin M. Dooley B. Cotter A. . (2006). Factor structures of measures of cognitive distortions, emotional congruence and victim empathy based on data from Irish child sex offenders. Ir. J. Psychol. 27, 142–149. doi: 10.1080/03033910.2006.10446237

[ref58] Wells G. A. Shea B. O'Connell D. Peterson J. Welch V. Tugwell P. (2011). The Newcastle-Ottawa scale (NOS) for assessing the quality of nonrandomized studies in meta-analysis Available online at: http://www.ohri.ca/programs/clinical_epidemiology/oxford.asp

[ref59] Whiting P. F. Rutjes A. W. Westwood M. E. Mallett S. Deeks J. J. Reitsma J. B. . (2011). QUADAS-2: a revised tool for the quality assessment of diagnostic accuracy studies. Ann. Intern. Med. 155, 529–536. doi: 10.7326/0003-4819-155-8-201110180-00009, 22007046

[ref60] World Health Organization (2024) Child maltreatment Available online at: https://www.who.int/news-room/fact-sheets/detail/child-maltreatment (accessed July 7, 2026)

[ref61] Yang F. (2022) Assessments and treatments for pedophilic disorder: a literature review International Conference on Biotechnology, Life Science and Medical Engineering (BLSME 2022), Jeju, South Korea

