# Supplementary material for: Assessing paedophilic sexual preferences in child sexual offenders using eye-tracking methods: an exploratory meta-analysis
Source: Front Psychol. 2026 Sep 8;17:1908178. doi: 10.3389/fpsyg.2026.1908178 (PMC13593482; doi:10.3389/fpsyg.2026.1908178)
Supplement: Supplementary file 1 [file Data_Sheet_1.docx]

Supplementary Material

1. **Appendix I: Full search terms MEDLINE (PubMed)**

Search August 24, 2023

| Search | Query | Records retrieved |
| --- | --- | --- |
| S1 | ("marker"[All Fields] OR "markers"[All Fields] OR "biomarker*"[All Fields] OR ("eye tracking technology"[MeSH Terms] OR ("eye tracking"[All Fields] AND "technology"[All Fields]) OR "eye tracking technology"[All Fields] OR ("eye"[All Fields] AND "tracking"[All Fields]) OR "eye tracking"[All Fields]) OR ("magnetic resonance imaging"[MeSH Terms] OR ("magnetic"[All Fields] AND "resonance"[All Fields] AND "imaging"[All Fields]) OR "magnetic resonance imaging"[All Fields] OR "fmri"[All Fields]) OR (("brain"[MeSH Terms] OR "brain"[All Fields] OR "brains"[All Fields] OR "brain s"[All Fields]) AND ("activable"[All Fields] OR "activate"[All Fields] OR "activated"[All Fields] OR "activates"[All Fields] OR "activating"[All Fields] OR "activation"[All Fields] OR "activations"[All Fields] OR "activator"[All Fields] OR "activator s"[All Fields] OR "activators"[All Fields] OR "active"[All Fields] OR "actived"[All Fields] OR "actively"[All Fields] OR "actives"[All Fields] OR "activities"[All Fields] OR "activity s"[All Fields] OR "activitys"[All Fields] OR "motor activity"[MeSH Terms] OR ("motor"[All Fields] AND "activity"[All Fields]) OR "motor activity"[All Fields] OR "activity"[All Fields])) OR ("neuroimaging"[MeSH Terms] OR "neuroimaging"[All Fields] OR ("brain"[All Fields] AND "imaging"[All Fields]) OR "brain imaging"[All Fields]) OR "neuroimag*"[All Fields] OR (("skin"[MeSH Terms] OR "skin"[All Fields]) AND ("behavior"[MeSH Terms] OR "behavior"[All Fields] OR "conduct"[All Fields] OR "conducting"[All Fields] OR "conducts"[All Fields] OR "conductance"[All Fields] OR "conductances"[All Fields] OR "conducted"[All Fields] OR "conductibility"[All Fields] OR "conduction"[All Fields] OR "conductions"[All Fields] OR "conductive"[All Fields] OR "conductively"[All Fields] OR "conductivities"[All Fields] OR "electric conductivity"[MeSH Terms] OR ("electric"[All Fields] AND "conductivity"[All Fields]) OR "electric conductivity"[All Fields] OR "conductivity"[All Fields])) OR (("heartrate"[All Fields] OR "heartrates"[All Fields]) AND ("variabilities"[All Fields] OR "variability"[All Fields] OR "variable"[All Fields] OR "variable s"[All Fields] OR "variables"[All Fields] OR "variably"[All Fields])) OR ("wearability"[All Fields] OR "wearable"[All Fields] OR "wearables"[All Fields]) OR "temperat*"[All Fields] OR ("electroencephalography"[MeSH Terms] OR "electroencephalography"[All Fields] OR "eeg"[All Fields]) OR "plethysmogr*"[All Fields] OR "polygraph*"[All Fields]) | 4.190.229 |
| S2 | ("forens*"[All Fields] OR "crim*"[All Fields] OR "offen*"[All Fields] OR "prison*"[All Fields] OR "secur*"[All Fields] OR "pedoph*"[All Fields] OR "paraphil*"[All Fields] OR ("antisocial personality disorder"[MeSH Terms] OR ("antisocial"[All Fields] AND "personality"[All Fields] AND "disorder"[All Fields]) OR "antisocial personality disorder"[All Fields] OR "psychopathy"[All Fields] OR "psychopathies"[All Fields]) | 429.185 |
| S3 | ("psychiatr*"[All Fields] OR (("mental"[All Fields] OR "mentalities"[All Fields] OR "mentality"[All Fields] OR "mentalization"[MeSH Terms] OR "mentalization"[All Fields] OR "mentalizing"[All Fields] OR "mentalize"[All Fields] OR "mentalized"[All Fields] OR "mentally"[All Fields]) AND "illn*"[All Fields]) OR (("mental"[All Fields] OR "mentalities"[All Fields] OR "mentality"[All Fields] OR "mentalization"[MeSH Terms] OR "mentalization"[All Fields] OR "mentalizing"[All Fields] OR "mentalize"[All Fields] OR "mentalized"[All Fields] OR "mentally"[All Fields]) AND "disord*"[All Fields]) OR "psychopath*"[All Fields] OR "inpati*"[All Fields] OR "pedophil*"[All Fields] OR "paraphil*"[All Fields]) | 1.353.219 |
| S4 | S1 AND S2 AND S3 | 4.621 |
| S5 | Limiters: none | 4.621 |

2. **Appendix II: Search strategy Google Scholar (Publish or Perish)**

Search August 24, 2023

| Source | Query |
| --- | --- |
| Google Scholar (Publish or Perish) | Marker\|Biomarkerǀ“Eye tracking”\|FMRI\|“Brain activation”\|”Brain imaging”\|Neuroimaging\|”Skin conductance”\|”Heart Rate variability”\|Wearable\|Temperature\|EEG\|Plethysmography\|Polygraph Forensic\|Criminal\|Offender\|Prisoner  Psychiatry\|”Mental illness” |

3. **Appendix III: Full search terms updated search**

Search January 18, 2026

| Source | Query |
| --- | --- |
| MEDLINE (PubMed) | ("eye tracking technology"[MeSH Terms] OR ("eye tracking"[All Fields] AND "technology"[All Fields]) OR "eye tracking technology"[All Fields] OR ("eye"[All Fields] AND "tracking"[All Fields]) OR "eye tracking"[All Fields]) AND ("forens*"[All Fields] OR "crim*"[All Fields] OR "offen*"[All Fields] OR "prison*"[All Fields] OR "secur*"[All Fields] OR "pedoph*"[All Fields] OR "paraphil*"[All Fields] AND pedophil*"[All Fields] OR "paraphil*"[All Fields]) |
| Google Scholar (Publish or Perish) | “Eye tracking”  Forensic\|Criminal\|Offender\|Prisoner  Pedophilia\|Paraphilia |

4. **Appendix IV: Forest plots meta-analyses**

**Figure 2: Viewing time PCSO vs. HC – Adult stimuli**

*Note.* PCSO = Paedophile Child Sex Offenders; HC = Healthy Controls; Additional information concerning populations, stimuli and outcome measures. Vásquez-Amézquita et al. (2019): PCSO (*N*=18) vs. HC (*N*=19), adult stimuli (data on full body male and female stimuli combined), outcome: total fixation time; Ilyina et al. (2022): PCSO (*N* = 10) vs. HC (*N* = 26), adult female stimuli (no adult male stimuli used), outcome: total fixation time.

**Figure 3: Viewing time PCSO vs. HC – Child stimuli**

*Note.* PCSO = Paedophile Child Sex Offenders; HC = Healthy Controls; Additional information concerning populations, stimuli, and outcome measures. Vásquez-Amézquita et al. (2019): PCSO (*N* = 18) vs. HC (*N* = 19), child stimuli (data on full body boy and girl stimuli combined), outcome: total fixation time; Ilyina et al. (2022): PCSO (*N* = 10) vs. HC (*N* = 26), child stimuli (data on boy and girl stimuli combined), outcome: total duration of fixations; Vásquez-Amézquita et al. (2019): PCSO (*N* = 18) vs. HC (*N* = 19), child stimuli (data on full body boy and girl stimuli combined), outcome: total fixation time.

**Figure 4: Viewing time PCSO vs. No clinical signs of paedophilia – Adult stimuli**

*Note.* PCSO = Paedophile Child Sex Offenders; Additional information concerning populations, stimuli, and outcome measures. Kamenskov et al. (2020): PCSO (*N* = 43) vs. No clinical signs of paedophilia (*N* = 57), adult female stimuli (no adult male stimuli used), outcome: total viewing time; Vásquez-Amézquita et al. (2019): PCSO (*N* = 18) vs. SOAA (*N* = 16), adult stimuli (data on full body male and female stimuli combined), outcome: total fixation time.

**Figure 5: Viewing time PCSO vs. No clinical signs of paedophilia – Child stimuli**

*Note.* PCSO = Paedophile Child Sex Offenders; Additional information concerning populations, stimuli, and outcome measures. Kamenskov et al. (2020): PCSO (*N* = 43) vs. No clinical signs of paedophilia (*N* = 57), child stimuli (data on full body boy and girl stimuli combined), outcome: total viewing time; Vásquez-Amézquita et al. (2019): PCSO (*N* = 18) vs. SOAA (*N* = 16), child stimuli (data on full body boy and girl stimuli combined), outcome: total fixation time.

**Figure 6: Fixation latency PCSO vs. HC – Adult stimuli**

*Note.* PCSO = Paedophile Child Sex Offenders; HC = Healthy Controls; Additional information concerning populations, stimuli, and outcome measures. Pezzoli et al. (2021): PCSO (*N* = 16 ) vs. HC (*N*=16), adult stimuli (in PCSO ‘nonpreferred’ stimuli; in HC ‘preferred’ stimuli), outcome: response latency; Vásquez-Amézquita et al. (2019): PCSO (*N* = 18) vs. HC (*N* = 19), adult stimuli (data on full body male and female stimuli combined), outcome: fixation latency.

**Figure 7: Fixation latency PCSO vs. HC – Child stimuli**

*Note.* PCSO = Paedophile Child Sex Offenders; HC = Healthy Controls; Additional information concerning populations, stimuli, and outcome measures. Fromberger et al. (2012): PCSO (*N* = 22) vs. HC (*N* = 52), child stimuli, outcome: fixation latency; Pezzoli et al. (2021): PCSO (*N* = 16) vs. HC (*N* = 16), child stimuli (in PCSO ‘preferred’ stimuli; in HC ‘nonpreferred’ stimuli), outcome: response latency; Vásquez-Amézquita et al. (2019): PCSO (*N* = 18) vs. HC (*N* = 19), child stimuli (data on full body boy and girl stimuli combined), outcome: fixation latency.

**Figure 8: Fixation latency PCSO vs. SOAA – Child stimuli**

*Note.* PCSO = Paedophile Child Sex Offenders; SOAA = Sex Offenders Against Adults; Additional information concerning populations, stimuli, and outcome measures. Fromberger et al. (2012): PCSO (*N* = 22) vs. SOAA (*N* = 8), child stimuli, outcome: fixation latency; Vásquez-Amézquita et al. (2019): PCSO (*N* = 18) vs. SOAA (*N* = 16), child stimuli (data on full body boy and girl stimuli combined), outcome: fixation latency.

**Figure 9: Fixation latency PCSO vs. Offenders – Child stimuli**

*Note.* PCSO = Paedophile Child Sex Offenders; Additional information concerning populations, stimuli, and outcome measures. Fromberger et al. (2012): PCSO (*N* = 22) vs. SOAA (*N* = 8), child stimuli, outcome: fixation latency; Vásquez-Amézquita et al. (2019): PCSO (*N* = 18) vs. Offenders (Sex Offenders Against Adults [SOAA] (*N* = 16) and Nonsexual offenders (*N* = 18), total: *N* = 34), child stimuli (data on full body boy and girl stimuli combined), outcome: fixation latency.

**Figure 10: Fixation latency PCSO vs. No clinical signs of paedophilia – Adult stimuli**

*Note.* PCSO = Paedophile Child Sex Offenders; Additional information concerning populations, stimuli and outcome measures. Vásquez-Amézquita et al. (2019): PCSO (*N*=18) vs. SOAA (*N* = 16), total fixation time on adult stimuli (data on full body male and female stimuli combined), outcome: fixation latency; Kamenskov et al. (2020): PCSO (*N* = 43) vs. No clinical signs of paedophilia (*N* = 57), adult female stimuli (no adult male stimuli used), outcome: entry time.

**Figure 11: Fixation latency PCSO vs. No clinical signs of paedophilia – Child stimuli**

*Note.* PCSO = Paedophile Child Sex Offenders; Additional information concerning populations, stimuli and outcome measures. Fromberger et al. (2012): PCSO (*N* = 22) vs. SOAA (*N* = 8), child stimuli, outcome: fixation latency; Kamenskov et al. (2020): PCSO (*N* = 43) vs. No clinical signs of paedophilia (*N* = 57), child stimuli (data on full body boy and girl stimuli combined), outcome: entry time; Vásquez-Amézquita et al. (2019): PCSO (*N* = 18) vs. SOAA (*N* = 16), child stimuli (data on full body boy and girl stimuli combined), outcome: fixation latency.

**Figure 12: Viewing time PSCO – Adult vs. child stimuli**

*Note.* PCSO = Paedophile Child Sex Offenders; Additional information concerning populations, stimuli and outcome measures. Ilyina et al. (2022): PCSO (*N* = 10), total duration of fixations on adult female stimuli (no adult male stimuli used) vs. on child stimuli (data on boy and girl stimuli combined); Kamenskov et al. (2020): PCSO (*N* = 43), viewing time on normal stimuli (adult female stimuli) vs. on deviant stimuli (data on full body boy and girl stimuli combined); Vásquez-Amézquita et al. (2019): PCSO (*N*=18), total fixation time on adult stimuli (data on full body male and female stimuli combined) vs. on child stimuli (data on full body boy and girl stimuli combined).

**Figure 13: Fixation latency PSCO – Adult vs. child stimuli**

*Note.* PCSO = Paedophile Child Sex Offenders; Additional information concerning populations, stimuli and outcome measures. Fromberger et al. (2013): PCSO (*N* = 22), entry time on adult stimuli vs. on child stimuli; Kamenskov et al. (2020): PCSO (*N* = 43), entry time on normal stimuli (adult female stimuli) vs. on deviant stimuli (data on full body boy and girl stimuli combined); Pezzoli et al. (2021): PCSO (*N* = 16) response latency on (nonpreferred) adult stimuli vs. on (preferred) child stimuli; Vásquez-Amézquita et al. (2019): PCSO (*N*=18), fixation latency on adult stimuli (data on full body male and female stimuli combined) vs. on child stimuli (data on full body boy and girl stimuli combined)

5. **Appendix V: Risk of bias and applicability judgments**
1) **Fromberger et al. (2012)**

| **Domain 1: Patient/control selection** | |
| --- | --- |
| A. **Risk of bias** |  |
| **Describe methods of patient selection:**  *“Paedophilic subjects (22) were recruited at high-security, forensic-psychiatric hospitals. Inclusion criteria were a cross-validated diagnosis of pedophilia by two experienced clinicians and mandatory hospitalization under treatment order for a child-sexual-abuse offense (validated through forensic records). Exclusion criteria were an acute psychotic episode or substance abuse during the previous month, no agreement between the two clinicians in respect to the diagnosis of pedophilia, or incapability or refusal to sign informed consent.”* | |
| - **Was a consecutive or random sample of patients enrolled?**   A non-consecutive sample of patients was enrolled. | Yes/**No**/Unclear |
| - **Was a case-control design avoided?** | Yes/**No**/Unclear |
| - **Did the study avoid inappropriate exclusions?**   Of the 65 screened forensic patients, 35 had to be  excluded (three paedophiles and 32 non-paedophiles). | Yes/**No**/Unclear |
| **Describe methods of control selection:**  *“Forensic control subjects (8) were recruited at high-security, forensic-psychiatric hospitals. Inclusion criteria were the absence of a diagnosis of pedophilia (cross-validated by two experienced clinicians), no child-sexual-abuse offense, and mandatory hospitalization under treatment order for an adult-sexual-abuse offense (validated through forensic records).*  *Healthy subjects (52) were recruited via a notice posted on bulletin boards in Gottingen and on inquiry at a police officer school. Inclusion criteria were the absence of any psychiatric illnesses, deviant sexual fantasies or behavior (validated by an extensive psychiatric and sexual anamnesis conducted in a systematic oral interview about the case history of the subject by one experienced clinician).*  *Exclusion criteria were an acute psychotic episode or substance abuse during the previous month, no agreement between the two clinicians in respect to the diagnosis of pedophilia, or incapability or refusal to sign informed consent.”* | |
| - **Was a consecutive or random sample of controls enrolled?**   A non-consecutive sample of controls was enrolled. | Yes/**No**/Unclear |
| - **Did the study avoid inappropriate exclusions?**   None of the non-forensic healthy controls had to be excluded. | Yes/No/**Unclear** |
| - **Were the control series used in the study derived from the same population as the cases, and essentially would have been cases had the outcome been present?** | Yes/**No**/Unclear |
| - **Was the history of outcome mentioned (no history of disease = endpoint)?** | **Yes**/No/Unclear |
| - **Was there comparability of cases and controls based on the design or analysis?**   The study controlled for age, IQ, hospitalisation, and AOI size. | **Yes**/No/Unclear |
| **Could the selection of patients have introduced bias?**  **Could the selection of controls have introduced bias?** | RISK: LOW/**HIGH**/UNCLEAR  RISK: LOW/**HIGH**/UNCLEAR |
| B. **Concerns regarding applicability** |  |
| **Describe included patients (prior testing, presentation, intended use of index test, and setting):**  *“Paedophilic subjects had a cross-validated diagnosis of paedophilia by two experienced clinicians and a mandatory hospitalization under treatment order for a child-sexual-abuse offense (validated through forensic records). They resided in high-security forensic hospitals.”* | |
| **Is there concern that the included patients do not match the review question?** | CONCERN: **LOW**/HIGH/UNCLEAR |
| **Describe included controls (prior testing, presentation, intended use of index test, and setting):**  *“Forensic control subjects were recruited at high-security, forensic-psychiatric hospitals. Inclusion criteria were the absence of a diagnosis of pedophilia (cross-validated by two experienced clinicians), no child-sexual-abuse offense, and mandatory hospitalization under treatment order for an adult-sexual-abuse offense (validated through forensic records).*  *Healthy subjects were recruited via a notice posted on bulletin boards in Gottingen and on inquiry at a police officer school. Inclusion criteria were the absence of any psychiatric illnesses, deviant sexual fantasies or behavior (validated by an extensive psychiatric and sexual anamnesis conducted in a systematic oral interview about the case history of the subject by one experienced clinician).”* | |
| **Is there concern that the included controls do not match the review question?**  Healthy individuals were only recruited through a notice in Gottingen/inquiry at a police officer training school. We cannot determine if this group of healthy subjects is representative of the general population. | CONCERN: LOW/**HIGH**/UNCLEAR |
| **Domain 2: Index test(s)** | |
| A. **Risk of bias** |  |
| **Describe the index test and how it was conducted and interpreted:**  Fixation latency was measured after a visual stimulus was presented.  *“Eye movements were recorded over 64 trials. Before each trial, a fixation cross appeared on the*  *screen. Two pictures (either the picture of a girl combined with the picture of a woman, or the*  *picture of a boy combined with the picture of a man) were presented in opposing corners of the*  *screen. After each stimulus presentation, participants had to answer a question. At the beginning*  *of each experiment and after the rest, a calibration was performed that consisted of instructing*  *the participant to fixate on nine points on the display area.*  *Eye movements were measured using an SMI iView X RED eye tracker (SensoMotoric Instruments GmBH, Berlin, Germany) in combination with an iView X workstation by measuring the corneal reflection and dark pupil with a videobased infrared eye camera. To identify fixations and saccades, the raw eyemovement data were analyzed with BeGaze 3 (SensoMotoric Instruments GmbH, Berlin, Germany). If eye movements remained stable within a circular area of 1° of visual angle for at least 100 milliseconds, this was classified as a fixation.*  *Fixation latency was defined as the duration from stimulus onset to the first fixation within an AOI, representing a measure for initial orienting. Eye movements represent automatic attentional processes. This article examined whether eye movement techniques can distinguish between people with and without paedophilia. It showed differences in index test results between cases and controls.”* | |
| - **Were the index test results interpreted without knowledge of the results of the reference standard?**   Not applicable: Objective index test measurement. | Yes/No/Unclear/**Not applicable** |
| - **If a threshold was used, was it pre-specified?**   Not applicable: No threshold was used. | Yes/No/Unclear/**Not applicable** |
| **Could the conduct or interpretation of the index test have introduced bias?** | RISK: **LOW**/HIGH/UNCLEAR |
| B. **Concerns regarding applicability** |  |
| **Is there concern that the index test, its conduct, or interpretation differs from the review question?** | CONCERN: **LOW**/HIGH/UNCLEAR |
| **A. Risk of bias** |  |
| **Describe the index test and how it was conducted and interpreted:**  Viewing time was measured after a visual stimulus was presented.  *The time from stimulus onset to the completion of the second rating (sexual arousal rating) was measured without the participant’s knowledge to additionally assess viewing time.*  *Eye movements were recorded over 64 trials. Before each trial a fixation cross appeard on the screen. As soon as the participant had gazed at the fixation cross for at least 500 milliseconds, the next trial started automatically. Two pictures (either the picture of a girl combined with the picture of a woman, or the picture of a boy combined with the picture of a man) were presented in opposing corners of the screen. After each stimulus presentation, participants had to answer a question. At the beginning of each experiment and after the rest, a calibration was performed that consisted of instructing the participant to fixate on nine points on the display area.*  *Eye movements were measured using an SMI iView X RED eye tracker (SensoMotoric Instruments GmBH, Berlin, Germany) in combination with an iView X workstation by measuring the corneal reflection and dark pupil with a videobased infrared eye camera.*  *Relative fixation time was defined as the sum fixation duration of all fixations located in the space of the relevant AOI divided by the whole presentation time. Relative fixation time is a measure for the overall attention a specific AOI attracts*. *Eye movements represent automatic attentional processes.*  *This article examined whether eye movement techniques can distinguish between people with and without paedophilia. It showed differences in index test results between cases and controls.* | |
| - **Were the index test results interpreted without knowledge of the results of the reference standard?**   Not applicable: Objective index test measurement. | Yes/No/Unclear/**Not applicable** |
| - **If a threshold was used, was it pre-specified?**   Not applicable: No threshold was used. | Yes/No/Unclear/**Not applicable** |
| **Could the conduct or interpretation of the index test have**  **introduced bias?** | RISK: **LOW**/HIGH/UNCLEAR |
| **B. Concerns regarding applicability** |  |
| **Is there concern that the index test, its conduct, or interpretation differ from the review question?** | CONCERN: **LOW**/HIGH/UNCLEAR |
| **Domain 3: Reference standard** | |
| A. **Risk of bias** |  |
| **Describe the reference standard and how it was conducted and interpreted:**  *The reference standard was a cross-validated diagnosis of paedophilia by two experienced clinicians and a mandatory hospitalization under treatment order for a child-sexual-abuse offense (validated through forensic records).* | |
| - **Is the reference standard likely to correctly classify the target condition?** | **Yes**/No/Unclear |
| - **Were the reference standard results interpreted without knowledge of the results of the index test?** | **Yes**/No/Unclear |
| **Could the reference standard, its conduct, or its interpretation have introduced bias?** | RISK: **LOW**/HIGH/UNCLEAR |
| B. **Concerns regarding applicability** |  |
| **Is there concern that the target condition as defined by the reference standard does not match the review question?** | CONCERN: **LOW**/HIGH/UNCLEAR |
| **Domain 4: Flow and timing** | |
| A. **Risk of bias** |  |
| **Describe any patients who did not receive the index test(s) and/or reference standard or who were excluded from the 2x2 table (refer to flow diagram):**  Viewing-time data for three pedophiles, one forensic control, and four non-forensic controls could not be analyzed because of technical difficulties.  **Describe the time interval and any interventions between index test(s) and reference standard:**  No information about the time interval between the reference standard and the index test(s). In this study, the time interval can not be inappropriate since a diagnosis of paedophilia is static. | |
| - **Was there an appropriate interval between index test(s) and reference standard?** | **Yes**/No/Unclear |
| - **Did all patients receive a reference standard?** | **Yes**/No/Unclear |
| - **Did patients receive the same reference standard?** | **Yes**/No/Unclear |
| - **Were all patients included in the analysis?** | Yes/**No**/Unclear |
| - **Could the patient flow have introduced bias?** | RISK: **LOW**/HIGH/UNCLEAR |

2) **Pezzoli et al. (2021)**

| **Domain 1: Patient/control selection** | |
| --- | --- |
| A. **Risk of bias** |  |
| **Describe methods of patient selection:**  *“Patients (16 male SOC, diagnosed with paedophilia and admitting being or having ever been sexually interested in prepubertal children aged 10 or younger,) were recruited from an outpatient sexual behaviors clinic at the Royal Ottawa Mental Health Centre. Patients were either on bail, probation, or parole for contact, noncontact, or both forms of sexual offenses against children under age 16. Before testing, we coded pertinent information (i.e., basic demographics, diagnosis, and criminal history) from clinical records, and we administered a self-report version of the Screening Scale for Pedophilic Interests, Version 2 (SSPI-2) (38), a behavioral life history measure designed for SOC. Exclusion criteria were potential contraindications for tDCS, including prior head injury with loss of consciousness >5 min, neurologic diagnosis including epilepsy, prior medical conditions with central nervous system sequelae, metallic implants inside the head, electrical medical devices in the body, as well as visual and hearing impairments potentially interfering with testing.”* | |
| - **Was a consecutive or random sample of patients enrolled?**   A non-consecutive sample of patients was enrolled. | Yes/**No**/Unclear |
| - **Was a case-control design avoided?** | Yes/**No**/Unclear |
| - **Did the study avoid inappropriate exclusions?**   *“Seventeen patients volunteered to participate but had to*  *be screened out. Almost half of the volunteers who were screened out (n = 7/17) reported a history of traumatic brain injury with loss of consciousness >5 min, in line with the elevated prevalence of traumatic brain injury often observed among offenders.”*  It is not clear based on which exclusion criteria the other  10 volunteers were excluded. | Yes/**No**/Unclear |
| **Describe methods of control selection:**  *“Controls (16) were recruited from the community via online advertising.*  *Exclusion criteria for all participants were potential contraindications for tDCS, including prior head injury with loss of consciousness > 5 min, neurologic diagnosis including epilepsy, prior medical conditions with central nervous system sequelae, metallic implants inside the head, electrical medical devices in the body, as well as visual and hearing impairments potentially interfering with testing. Any criminal history (self-reported), a history of any clinically significant psychiatric condition in the last six months, and current psychoactive medication use constituted exclusion criteria.”* | |
| - **Was a consecutive or random sample of controls enrolled?** | Yes/**No**/Unclear |
| - **Did the study avoid inappropriate exclusions?** | Yes/No/**Unclear** |
| - **Were the control series used in the study derived from the same population as the cases, and essentially would have been cases had the outcome been present?** | Yes/**No**/Unclear |
| - **Was the history of outcome mentioned (no history of disease = endpoint)?** | **Yes**/No/Unclear |
| - **Was there comparability of cases and controls based on the design or analysis?**   The study controlled for age. Groups were matched on years of education, hand dominance, and overall attention. | **Yes**/No/Unclear |
| **Could the selection of patients have introduced bias?**  **Could the selection of controls have introduced bias?** | RISK: LOW/**HIGH**/UNCLEAR  RISK: LOW/**HIGH**/UNCLEAR |
| B. **Concerns regarding applicability** |  |
| **Describe included patients (prior testing, presentation, intended use of index test, and setting):**  *“Patients (16 male SOC, diagnosed with paedophilia and admitting being or having ever been*  *sexually interested in prepubertal children aged 10 or younger,) were recruited from an outpatient*  *sexual behaviors clinic at the Royal Ottawa Mental Health Centre. Patients were either on bail,*  *probation, or parole for contact, noncontact, or both forms of sexual offenses against children*  *under age 16. Before testing, we coded pertinent information (i.e., basic demographics, diagnosis,*  *and criminal history) from clinical records, and we administered a self-report version of the*  *Screening Scale for Pedophilic Interests, Version 2 (SSPI-2) (38), a behavioral life history*  *measure designed for SOC.”* | |
| **Is there concern that the included patients do not match the review question?** | CONCERN: **LOW**/HIGH/UNCLEAR |
| **Describe included controls (prior testing, presentation, intended use of index test, and setting):**  *“Controls (16) were recruited from the community via online advertising.*  *Exclusion criteria for all participants were potential contraindications for tDCS, including prior head injury with loss of consciousness > 5 min, neurologic diagnosis including epilepsy, prior medical conditions with central nervous system sequelae, metallic implants inside the head, electrical medical devices in the body, as well as visual and hearing impairments potentially interfering with testing. Any criminal history (self-reported), a history of any clinically significant psychiatric condition in the last six months, and current psychoactive medication use constituted exclusion criteria.”*  *There is insufficient information about the selection process to see whether the healthy subjects*  *group is representative of the general population. The study does not indicate whether any healthy*  *subjects were excluded from the analysis, nor does it clarify if they randomly selected 16 controls*  *from the volunteering participants.* | |
| **Is there concern that the included controls do not match the review question?** | CONCERN: LOW/**HIGH**/UNCLEAR |
| **Domain 2: Index test(s)** | |
| A. **Risk of bias** |  |
| **Describe the index test and how it was conducted and interpreted:**  Response latency was measured after a visual stimulus was presented.  *“During the antisaccade test, participants’ eye movements were recorded using a contactless remote-controlled eye tracking device (SMI RED 250 eye tracker; SensoMotoric Instruments GmBH, Germany). This device recorded the corneal reflection caused by an infrared light, stored coordinates of gaze position on the screen with 250 Hz frequency, automatically excluded blinking or off-screen gazes, and automatically compensated for minor head movements.*  *The antisaccade test included 202 trials. One practice block of 10 trials (neutral stimuli) was administered before tDCS. The remaining 192 test trials with sexually relevant stimuli from the VPS and NRP picture sets were administered during tDCS. During each trial, two images appeared on the computer monitor, one of a child and one of an adult. In prosaccade blocks, participants were instructed to look toward the stimulus surrounded by the visual cue. In antisaccade blocks, they had to look toward the opposite stimulus.*  *Response latency was defined as time from stimuli appearance to first fixation towards target.”* | |
| - **Were the index test results interpreted without knowledge of the results of the reference standard?**   Not applicable: Objective index test measurement. | Yes/No/Unclear/**Not applicable** |
| - **If a threshold was used, was it pre-specified?** | Yes/No/Unclear/**Not applicable** |
| **Could the conduct or interpretation of the index test have introduced bias?** | RISK: **LOW/**HIGH/UNCLEAR |
| B. **Concerns regarding applicability** |  |
| **Is there concern that the index test, its conduct, or interpretation differs from the review question?** | CONCERN: **LOW**/HIGH/UNCLEAR |
| **Domain 3: Reference standard** | |
| A. **Risk of bias** |  |
| **Describe the reference standard and how it was conducted and interpreted:**  *“Patients (16 male SOC, diagnosed with paedophilia and admitting being or having ever been sexually interested in prepubertal children aged 10 or younger,) were recruited from an outpatient sexual behaviors clinic at the Royal Ottawa Mental Health Centre. Patients were either on bail, probation, or parole for contact, noncontact, or both forms of sexual offenses against children under age 16. Before testing, we coded pertinent information (i.e., basic demographics, diagnosis, and criminal history) from clinical records, and we administered a self-report version of the Screening Scale for Pedophilic Interests, Version 2 (SSPI-2) (38), a behavioral life history measure designed for SOC.”*  The text does not specify the methods used for diagnosing the patients. However, the researchers indicate that the individuals have been diagnosed with pedophilia, are receiving treatment at an outpatient sexual behaviour clinic, and are currently on bail, probation, or parole for sexual offenses against children under the age of 16. Furthermore, these individuals have self-reported their sexual interests in children. | |
| - **Is the reference standard likely to correctly classify the target condition?** | **Yes**/No/Unclear |
| - **Were the reference standard results interpreted without knowledge of the results of the index test?** | **Yes**/No/Unclear |
| **Could the reference standard, its conduct, or its interpretation have introduced bias?** | RISK: **LOW**/HIGH/UNCLEAR |
| B. **Concerns regarding applicability** |  |
| **Is there concern that the target condition as defined by the reference standard does not match the review question?** | CONCERN: **LOW**/HIGH/UNCLEAR |
| **Domain 4: Flow and timing** | |
| A. **Risk of bias** |  |
| **Describe any patients who did not receive the index test(s) and/or reference standard or who were excluded from the 2x2 table (refer to flow diagram):**  /  **Describe the time interval and any interventions between index test(s) and reference standard:**  No information about the time interval between the reference standard and the index test(s). In this study, the time interval can not be inappropriate since a diagnosis of paedophilia is static. | |
| - **Was there an appropriate interval between index test(s) and reference standard?** | **Yes**/No/Unclear |
| - **Did all patients receive a reference standard?** | **Yes**/No/Unclear |
| - **Did patients receive the same reference standard?** | **Yes**/No/Unclear |
| - **Were all patients included in the analysis?** | **Yes**/No/Unclear |
| **Could the patient flow have introduced bias?** | RISK: **LOW**/HIGH/UNCLEAR |

3) **Vásquez-Amézquita et al. (2019)**

| **Domain 1: Patient/control selection** | |
| --- | --- |
| A. **Risk of bias** |  |
| **Describe methods of patient selection:**  *“Patients were convicted or sentenced for “abusive carnal access with a minor under 14 years of age”. Participants were selected from men who had a victim under the age of 12. Due to the absence of a qualified professional, no accurate assessment of paedophilia based on a forensic psychiatric evaluation was made. Moreover, prison files did not contain sufficient information to assess pedophilic sexual interests. Thus, the Children and Sex Cognitive Distortions Scale (Waldron et al., 2006) was applied, of which we used seven items as selection criteria for the group of sexual offenders against children. These seven items were chosen based on the assessment of four local psychologists that they were relevant to sexual interests toward children. We selected sexual offenders against children with a score of 3 or 4 (which ranged from 0 = Very false to 4 = Very true) on each of these seven items (N = 18).”* | |
| - **Was a consecutive or random sample of patients enrolled?**   A non-consecutive sample of patients was enrolled. | Yes/**No**/Unclear |
| - **Was a case-control design avoided?** | Yes/**No**/Unclear |
| - **Did the study avoid inappropriate exclusions?** | **Yes**/No/Unclear |
| **Describe methods of control selection:**  *“Forensic control groups were recruited in the same prison as the paedophilic subjects group, while non-offending controls were recruited from security personnel from private companies in the city. Control groups had scores of 0 or 1 on the seven items of the Children and Sex Cognitive Distortions Scale (Waldron et al., 2006).”* | |
| - **Was a consecutive or random sample of controls enrolled?**   A non-consecutive sample of controls was enrolled. | Yes/**No**/Unclear |
| - **Did the study avoid inappropriate exclusions?**. | **Yes**/No/Unclear |
| - **Were the control series used in the study derived from the same population as the cases, and essentially would have been cases had the outcome been present?** | Yes/**No**/Unclear |
| - **Was the history of outcome mentioned (no history of disease = endpoint)?** | **Yes**/No/Unclear |
| - **Was there comparability of cases and controls based on the design or analysis?**   All three offender groups were similar in age and education level. | **Yes**/No/Unclear |
| **Could the selection of patients have introduced bias?**  **Could the selection of controls have introduced bias?** | RISK: LOW/**HIGH**/UNCLEAR  RISK: LOW/**HIGH**/UNCLEAR |
| B. **Concerns regarding applicability** |  |
| **Describe included patients (prior testing, presentation, intended use of index test, and setting):**  *“Patients were convicted or sentenced for “abusive carnal access with a minor under 14 years of age”. Participants were selected from men who had a victim under the age of 12. Due to the absence of a qualified professional, no accurate assessment of paedophilia based on a forensic psychiatric evaluation was made. Moreover, prison files did not contain sufficient information to assess pedophilic sexual interests. Thus, the Children and Sex Cognitive Distortions Scale (Waldron et al., 2006) was applied, of which we used seven items as selection criteria for the group of sexual offenders against children. These seven items were chosen based on the assessment of four local psychologists that they were relevant to sexual interests toward children. We selected sexual offenders against children with a score of 3 or 4 (which ranged from 0 = Very false to 4 = Very true) on each of these seven items (N = 18).”* | |
| **Is there concern that the included patients do not match the review question?**  Although the patients were convicted or sentenced for offenses against minors and scored high on sexual cognitive distortions scales (which is associated with paedophilia), the researchers did not perform an officially validated diagnosis of paedophilia. However, because of all the stated facts and associations with paedophilia, we can assume that the included patients to match the review question. | CONCERN: **LOW**/HIGH/UNCLEAR |
| **Describe included controls (prior testing, presentation, intended use of index test, and setting):**  *“Forensic control groups were recruited in the same prison as the paedophilic subjects group, while non-offending controls were recruited from security personnel from private companies in the city. Control groups had scores of 0 or 1 on the seven items of the Children and Sex Cognitive Distortions Scale (Waldron et al., 2006).”* | |
| **Is there concern that the included controls do not match the review question?**  Healthy individuals were recruited only from security personnel. We cannot determine if this group of healthy subjects is representative of the general population. | CONCERN: LOW/**HIGH**/UNCLEAR |
| **Domain 2: Index test(s)** | |
| A. **Risk of bias** |  |
| **Describe the index test and how it was conducted and interpreted:**  Fixation latency was measured after a visual stimulus was presented.  *The experiment consisted of 64 trials containing images of adults and children; each trial displayed a pair of images depicting people who differed in age (adult versus child) but not sex.*  *For recording of eye movements and analysis of fixations, we used the Tobii Studio Eye Tracker, Tobii Pro X2-60 integrated with the Tobii Studio Pro Version 3.3.2 (Tobii Technology AB, Stockholm, Sweden).*  *The number of first fixations and the time to first fixation on an area of interest were used as indicators of early attention.* | |
| - **Were the index test results interpreted without knowledge of the results of the reference standard?**   Not applicable: Objective index test measurement. | Yes/No/Unclear/**Not applicable** |
| - **If a threshold was used, was it pre-specified?**   Not applicable: No threshold was used. | Yes/No/Unclear/**Not applicable** |
| **Could the conduct or interpretation of the index test have introduced bias?** | RISK: **LOW**/HIGH/UNCLEAR |
| B. **Concerns regarding applicability** |  |
| **Is there concern that the index test, its conduct, or interpretation differs from the review question?** | CONCERN: **LOW**/HIGH/UNCLEAR |
| **A. Risk of bias** |  |
| **Describe the index test and how it was conducted and interpreted:**  Viewing time was measured after a visual stimulus was presented.  *The experiment consisted of 64 trials containing images of adults and children; each trial displayed a pair of images depicting people who differed in age (adult versus child) but not sex.*  *For recording of eye movements and analysis of fixations, we used the Tobii Studio Eye Tracker, Tobii Pro X2-60 integrated with the Tobii Studio Pro Version 3.3.2 (Tobii Technology AB, Stockholm, Sweden).*  *For the measurement of late attentional processes, we used the total duration of fixations, which is the average of the total amount of time spent looking at an area of interest, as well as the fixation count.* | |
| - **Were the index test results interpreted without knowledge of the results of the reference standard?**   Not applicable: Objective index test measurement. | Yes/No/Unclear/**Not applicable** |
| - **If a threshold was used, was it pre-specified?**   Not applicable: No threshold was used. | Yes/No/Unclear/**Not applicable** |
| **Could the conduct or interpretation of the index test have**  **introduced bias?** | RISK: **LOW**/HIGH/UNCLEAR |
| **B. Concerns regarding applicability** |  |
| **Is there concern that the index test, its conduct, or interpretation differ from the review question?** | CONCERN: **LOW**/HIGH/UNCLEAR |
| **Domain 3: Reference standard** | |
| A. **Risk of bias** |  |
| **Describe the reference standard and how it was conducted and interpreted:**  *“Due to the absence of a qualified professional, no accurate assessment of paedophilia based on a forensic psychiatric evaluation was made. Moreover, prison files did not contain sufficient information to assess pedophilic sexual interests. Thus, the Children and Sex Cognitive Distortions Scale (Waldron et al., 2006) was applied, of which we used seven items as selection criteria for the group of sexual offenders against children. These seven items were chosen based on the assessment of four local psychologists that they were relevant to sexual interests toward children. We selected sexual offenders against children with a score of 3 or 4 (which ranged from 0 = Very false to 4 = Very true) on each of these seven items (N = 18).”* | |
| - **Is the reference standard likely to correctly classify the target condition?**   Although the patients were convicted or sentenced for offenses against minors and scored high on sexual cognitive distortions scales (which is associated with paedophilia), the researchers did not perform an officially validated diagnosis of paedophilia. However, we can assume that the reference standard correctly classifies the targer condition. | **Yes**/No/Unclear |
| - **Were the reference standard results interpreted without knowledge of the results of the index test?** | **Yes**/No/Unclear |
| **Could the reference standard, its conduct, or its interpretation have introduced bias?** | RISK: **LOW**/HIGH/UNCLEAR |
| B. **Concerns regarding applicability** |  |
| **Is there concern that the target condition as defined by the reference standard does not match the review question?** | CONCERN: **LOW**/HIGH/UNCLEAR |
| **Domain 4: Flow and timing** | |
| A. **Risk of bias** |  |
| **Describe any patients who did not receive the index test(s) and/or reference standard or who were excluded from the 2x2 table (refer to flow diagram):**  /  **Describe the time interval and any interventions between index test(s) and reference standard:**  No information about the time interval between the reference standard and the index test(s). In this study, the time interval can not be inappropriate since a diagnosis of paedophilia is static. | |
| - **Was there an appropriate interval between index test(s) and reference standard?** | **Yes**/No/Unclear |
| - **Did all patients receive a reference standard?** | **Yes**/No/Unclear |
| - **Did patients receive the same reference standard?** | **Yes**/No/Unclear |
| - **Were all patients included in the analysis?** | **Yes**/No/Unclear |
| **Could the patient flow have introduced bias?** | RISK: **LOW**/HIGH/UNCLEAR |

4) **Ilyina et al. (2022)**

| **Domain 1: Patient/control selection** | |
| --- | --- |
| A. **Risk of bias** |  |
| **Describe methods of patient selection:**  *“Patients were selected in the Laboratory of Forensic Sexology of the Serbsky National Medical Research Center for Sexual Addiction of the Ministry of Health of the Russian Federation. Subjects with a diagnosis of paedophila and who were prosecuted for committing crimes against sexual inviolability of children were examined (N = 10). Diagnosis was made based on the results of a comprehensive evaluation of the sexological and psychiatric examination.”* | |
| - **Was a consecutive or random sample of patients enrolled?**   A non-consecutive sample of patients was enrolled. | Yes/**No**/Unclear |
| - **Was a case-control design avoided?** | Yes/**No**/Unclear |
| - **Did the study avoid inappropriate exclusions?**   The exclusion criteria are presented in the article. However, the researchers do not specify how many participants were excluded and which participant was excluded for which reason. | Yes/No/**Unclear** |
| **Describe methods of control selection:**  *“Control group: No clinical signs of sexual preference disorders.”* | |
| - **Was a consecutive or random sample of controls enrolled?**   A non-consecutive sample of controls was enrolled. | Yes/**No**/Unclear |
| - **Did the study avoid inappropriate exclusions?**   The exclusion criteria are presented in the article. However, the researchers do not specify how many participants were excluded and which participant was excluded for which reason. | Yes/No/**Unclear** |
| - **Were the control series used in the study derived from the same population as the cases, and essentially would have been cases had the outcome been present?** | Yes/**No**/Unclear |
| - **Was the history of outcome mentioned (no history of disease = endpoint)?** | **Yes**/No/Unclear |
| - **Was there comparability of cases and controls based on the design or analysis?** | Yes/No/**Unclear** |
| **Could the selection of patients have introduced bias?**  **Could the selection of controls have introduced bias?** | RISK: LOW/**HIGH**/UNCLEAR  RISK: LOW/HIGH/**UNCLEAR** |
| B. **Concerns regarding applicability** |  |
| **Describe included patients (prior testing, presentation, intended use of index test, and setting):**  *“Patients were selected in the Laboratory of Forensic Sexology of the Serbsky National Medical Research Center for Sexual Addiction of the Ministry of Health of the Russian Federation. Sub-experts with a diagnosis of paedophila and who were prosecuted for committing crimes against sexual inviolability of children were examined (N = 10). Diagnosis was made based on the results of a comprehensive evaluation of the sexological and psychiatric examination.”* | |
| **Is there concern that the included patients do not match the review question?** | CONCERN: **LOW**/HIGH/UNCLEAR |
| **Describe included controls (prior testing, presentation, intended use of index test, and setting):**  *“Control group: No clinical signs of sexual preference disorders.”* | |
| **Is there concern that the included controls do not match the review question?** | CONCERN: LOW/HIGH/**UNCLEAR** |
| **Domain 2: Index test(s)** | |
| A. **Risk of bias** |  |
| **Describe the index test and how it was conducted and interpreted:**  Viewing time was measured after a visual stimulus was presented.  *“Eye movements were measured and analyzed and the direction of the gaze was determined. At the beginning of the study a calibration of the gaze was carried out. 30 static images of dressed women with children (boy or girl) in the everyday environment were presented Each slide was shown 3 seconds. To study the specifics of viewing stimuli and quantitative analysis on each image, zones of interest were distinguished (boy/girl/woman) and the total duration of fixations and the number of fixations in each zone were estimated.”* | |
| - **Were the index test results interpreted without knowledge of the results of the reference standard?**   Not applicable: Objective index test measurement. | Yes/No/Unclear/**Not applicable** |
| - **If a threshold was used, was it pre-specified?**   Not applicable: No threshold was used. | Yes/No/Unclear/**Not applicable** |
| **Could the conduct or interpretation of the index test have introduced bias?** | RISK: **LOW**/HIGH/UNCLEAR |
| B. **Concerns regarding applicability** |  |
| **Is there concern that the index test, its conduct, or interpretation differs from the review question?** | CONCERN: **LOW**/HIGH/UNCLEAR |
| **Domain 3: Reference standard** | |
| A. **Risk of bias** |  |
| **Describe the reference standard and how it was conducted and interpreted:**  *“Diagnosis was made based on the results of a comprehensive evaluation of the sexological and psychiatric examination.”* | |
| - **Is the reference standard likely to correctly classify the target condition?** | **Yes**/No/Unclear |
| - **Were the reference standard results interpreted without knowledge of the results of the index test?** | **Yes**/No/Unclear |
| **Could the reference standard, its conduct, or its interpretation have introduced bias?** | RISK: **LOW**/HIGH/UNCLEAR |
| B. **Concerns regarding applicability** |  |
| **Is there concern that the target condition as defined by the reference standard does not match the review question?** | CONCERN: **LOW**/HIGH/UNCLEAR |
| **Domain 4: Flow and timing** | |
| A. **Risk of bias** |  |
| **Describe any patients who did not receive the index test(s) and/or reference standard or who were excluded from the 2x2 table (refer to flow diagram):**  No information available.  **Describe the time interval and any interventions between index test(s) and reference standard:**  No information about the time interval between the reference standard and the index test(s). In this study, the time interval can not be inappropriate since a diagnosis of paedophilia is static. | |
| - **Was there an appropriate interval between index test(s) and reference standard?** | **Yes**/No/Unclear |
| - **Did all patients receive a reference standard?** | **Yes**/No/Unclear |
| - **Did patients receive the same reference standard?** | **Yes**/No/Unclear |
| - **Were all patients included in the analysis?** | Yes/No/**Unclear** |
| **Could the patient flow have introduced bias?** | RISK: **LOW**/HIGH/UNCLEAR |

5) **Kamenskov et al. (2020)**

| **Domain 1: Patient/control selection** | |
| --- | --- |
| A. **Risk of bias** |  |
| **Describe methods of patient selection:**  *“The present empirical study was conducted at the National Medical Research Center for Psychiatry and Narcology named after V.P. Serbsky. The patient group consisted of 43 individuals with sexual preference disorders, including heterosexual pedophilia (ICD-10 category F65.4) (56%), homosexual pedophilia (F65.4) (25%), and bisexual pedophilia (F65.4) (19%). Inclusion criteria for the main group were a clinical diagnosis of pedophilia according to ICD-10 section F65.4. All subjects were accused of sexual acts against prepubertal and early pubertal children. The criteria for exclusion were the presence of psychotic symptoms, severe visual impairment requiring correction, and acute somatic or infectious diseases.”* | |
| - **Was a consecutive or random sample of patients enrolled?**   A non-consecutive sample of patients was enrolled. | Yes/**No**/Unclear |
| - **Was a case-control design avoided?** | Yes/**No**/Unclear |
| - **Did the study avoid inappropriate exclusions?**   No information about excluded participants. | Yes/No/**Unclear** |
| **Describe methods of control selection:**  *“The control group consisted of 57 individuals without signs of sexual preference disorders: both volunteers (21) and subjects who had committed sexual offences but without clinical signs of paraphilia (36). These individuals were grouped together because they were similar in their sexuality, which was consistent with the norm. In both cases, the subjects demonstrated the development of a heterosexual orientation, characterized by persistent platonic-erotic sexual attraction to women and its realization. Analysis of the psychophysiological experiment data also revealed no differences in the nature of viewing stimulus material between volunteers and offenders without sexual preference disorders. A similar approach (grouping volunteers and offenders without paraphilias) has been used by other researchers, who also found no significant differences between the two cohorts. The criteria for exclusion were the presence of psychotic symptoms, severe visual impairment requiring correction, and acute somatic or infectious diseases.”* | |
| - **Was a consecutive or random sample of controls enrolled?**   A non-consecutive sample of controls was enrolled. | Yes/**No**/Unclear |
| - **Did the study avoid inappropriate exclusions?**   No information about excluded participants. | Yes/No/**Unclear** |
| - **Were the control series used in the study derived from the same population as the cases, and essentially would have been cases had the outcome been present?** | Yes/**No**/Unclear |
| - **Was the history of outcome mentioned (no history of disease = endpoint)?** | **Yes**/No/Unclear |
| - **Was there comparability of cases and controls based on the design or analysis?** | Yes/No/**Unclear** |
| **Could the selection of patients have introduced bias?**  **Could the selection of controls have introduced bias?** | RISK: LOW/**HIGH**/UNCLEAR  RISK: LOW/**HIGH**/UNCLEAR |
| B. **Concerns regarding applicability** |  |
| **Describe included patients (prior testing, presentation, intended use of index test, and setting):**  *“The present empirical study was conducted at the National Medical Research Center for Psychiatry and Narcology named after V.P. Serbsky. The patient group consisted of 43 individuals with sexual preference disorders, including heterosexual pedophilia (ICD-10 category F65.4) (56%), homosexual pedophilia (F65.4) (25%), and bisexual pedophilia (F65.4) (19%). Inclusion criteria for the main group were a clinical diagnosis of pedophilia according to ICD-10 section F65.4. All subjects were accused of sexual acts against prepubertal and early pubertal children.”* No information is given about the recruitment setting. | |
| **Is there concern that the included patients do not match the review question?** | CONCERN: **LOW**/HIGH/UNCLEAR |
| **Describe included controls (prior testing, presentation, intended use of index test, and setting):**  *“The control group consisted of 57 individuals without signs of sexual preference disorders: both volunteers (21) and subjects who had committed sexual offences but without clinical signs of paraphilia (36). These individuals were grouped together because they were similar in their sexuality, which was consistent with the norm. In both cases, the subjects demonstrated the development of a heterosexual orientation, characterised by persistent platonic-erotic sexual attraction to women and its realisation.”*  No information is given about the recruitment setting. | |
| **Is there concern that the included controls do not match the review question?**  No information is provided about the recruitment settings for either the volunteers or the forensic controls. Healthy controls and forensic controls have been combined, making it unclear whether this control group is representative of the review question. | CONCERN: LOW/**HIGH**/UNCLEAR |
| **Domain 2: Index test(s)** | |
| A. **Risk of bias** |  |
| **Describe the index test and how it was conducted and interpreted:**  Fixation latency was measured after a visual stimulus was presented and was defined *“as gaze entry time into the region (the time from the onset of stimulus presentation until gaze enters the GI).”*  *“A high-speed remote binocular tracking system (SMIiViewXRED (Germany) in combination with the iView workstation) was used for eye tracking. Eye movements are recorded using an infrared camera, which also records the corneal reflex and pupil dilation/constriction. Erotic stimuli were presented on an LCD monitor positioned 60-70 cm from the eyes. Photographs of nude models—women (normative heterosexual stimuli) and children of both sexes (deviant stimuli)—were used in the experiment.”* | |
| - **Were the index test results interpreted without knowledge of the results of the reference standard?**   Not applicable: Objective index test measurement. | Yes/No/Unclear/**Not applicable** |
| - **If a threshold was used, was it pre-specified?**   Not applicable: No threshold was used. | Yes/No/Unclear/**Not applicable** |
| **Could the conduct or interpretation of the index test have**  **introduced bias?** | RISK: **LOW**/HIGH/UNCLEAR |
| B. **Concerns regarding applicability** |  |
| **Is there concern that the index test, its conduct, or interpretation differs from the review question?** | CONCERN: **LOW**/HIGH/UNCLEAR |
| A. **Risk of bias** |  |
| **Describe the index test and how it was conducted and interpreted:**  Viewing time was measured after a visual stimulus was presented and was defined *“as total stimulus viewing time (the sum of the durations of all saccades and fixations in the GI”.*  *“A high-speed remote binocular tracking system (SMIiViewXRED (Germany) in combination with the iView workstation) was used for eye tracking. Eye movements are recorded using an infrared camera, which also records the corneal reflex and pupil dilation/constriction. Erotic stimuli were presented on an LCD monitor positioned 60-70 cm from the eyes. Photographs of nude models—women (normative heterosexual stimuli) and children of both sexes (deviant stimuli)—were used in the experiment.”* | |
| - **Were the index test results interpreted without knowledge of the results of the reference standard?**   Not applicable: Objective index test measurement. | Yes/No/Unclear/**Not applicable** |
| - **If a threshold was used, was it pre-specified?**   Not applicable: No threshold was used. | Yes/No/Unclear/**Not applicable** |
| **Could the conduct or interpretation of the index test have**  **introduced bias?** | RISK: **LOW**/HIGH/UNCLEAR |
| B. **Concerns regarding applicability** |  |
| **Is there concern that the index test, its conduct, or interpretation differs from the review question?** | CONCERN: **LOW**/HIGH/UNCLEAR |
| **Domain 3: Reference standard** | |
| A. **Risk of bias** |  |
| **Describe the reference standard and how it was conducted and interpreted:**  *“The patient group consisted of 43 individuals with sexual preference disorders, including heterosexual pedophilia (ICD-10 category F65.4) (56%), homosexual pedophilia (F65.4) (25%), and bisexual pedophilia (F65.4) (19%). Inclusion criteria for the main group were a clinical diagnosis of pedophilia according to ICD-10 section F65.4.”*  No further information is provided about the diagnostic process. | |
| - **Is the reference standard likely to correctly classify the target condition?** | **Yes**/No/Unclear |
| - **Were the reference standard results interpreted without knowledge of the results of the index test?** | **Yes**/No/Unclear |
| **Could the reference standard, its conduct, or its interpretation have introduced bias?** | RISK: **LOW**/HIGH/UNCLEAR |
| B. **Concerns regarding applicability** |  |
| **Is there concern that the target condition as defined by the reference standard does not match the review question?** | CONCERN: **LOW**/HIGH/UNCLEAR |
| **Domain 4: Flow and timing** | |
| A. **Risk of bias** |  |
| **Describe any patients who did not receive the index test(s) and/or reference standard or who were excluded from the 2x2 table (refer to flow diagram):**  No information is given about excluded participants.  **Describe the time interval and any interventions between index test(s) and reference standard:**  No information about the time interval between the reference standard and the index test(s). In this study, the time interval can not be inappropriate since a diagnosis of paedophilia is static. | |
| - **Was there an appropriate interval between index test(s) and reference standard?** | **Yes**/No/Unclear |
| - **Did all patients receive a reference standard?** | **Yes**/No/Unclear |
| - **Did patients receive the same reference standard?** | **Yes**/No/Unclear |
| - **Were all patients included in the analysis?** | Yes/No/**Unclear** |
| **Could the patient flow have introduced bias?** | RISK: **LOW**/HIGH/UNCLEAR |

6. **Appendix VI: Raw data of the included studies**

**Supplementary Table 1: List of Raw Data Pezzoli et al. (2021)**

|  | ***M*_1_** | ***M*_2_** | ***SD*_1_** | ***SD*_2_** | ***Pooled SD*** | **Hedges’ g** | **Factor J** | **Hedges’ g (corrected)** | **SE_g_** |
| --- | --- | --- | --- | --- | --- | --- | --- | --- | --- |
| Fixation latency PCSO vs. HC – Adult stimuli | 354.73 | 405.27 | 68.09 | 89.09 | 79.2883 | 0.6374 | 0.9748 | -0.6213 | 0.3620 |
| Fixation latency PCSO vs. HC – Child stimuli | 345.68 | 383.58 | 92.31 | 73.22 | 83.3136 | -0.4549 | 0.9748 | -0.4434 | 0.3579 |
| Fixation latency PCSO – Adult vs. child stimuli | 354.73 | 345.68 | 68.09 | 92.31 | 81.1091 | 0.1116 | 0.9748 | 0.1088 | 0.3538 |

Note: *M*_1,_ *SD*_1_= mean and standard deviation of PCSO. *M*_2,_ *SD*_2_ *= mean and standard deviation of the control sample.* *Formulas used:* ***Pooled SD*** = √[(*n*_1_ - 1)**SD*_1_^2^ + (*n*_2_ - 1)**SD*_2_^2^] / (*n*_1_ + *n*_2_ - 2). ***g*** = (*M*_1_ - *M*_2_) / √[(*n*_1_ - 1)**SD*_1_^2^ + (*n*_2_ - 1)**SD*_2_^2^] / (*n*_1_ + *n*_2_ - 2). Since N < 50 (*n*_1_ PCSO = 16 + *n*_2_ HC = 16), g was multiplied by a correction **factor J** = 1-3/(4*N*-9). **SE_g_** = √[(*n*_1_ + *n*_2_) / *n*_1_**n*_2_ + *g*^2^ / 2(*n*_1_ + *n*_2_)].

**Supplementary Table 2: List of Raw Data Ilyina et al. (2022)**

|  | ***M*_1_** | ***M*_2_** | **SE_1_** | **SE_2_** | ***SD*_1_** | ***SD*_2_** | ***Pooled SD*** | **Hedges’ g** | **Factor J** | **Hedges’ g (corrected)** | **SE_g_** |
| --- | --- | --- | --- | --- | --- | --- | --- | --- | --- | --- | --- |
| Viewing time PCSO vs. HC – Adult stimuli | 987 | 988 | 32.8 | 19.4 | 103.7227 | 98.9210 | 100.2144 | -0.0098 | 0.9778 | -0.0096 | 0.3721 |
| Viewing time PCSO vs. HC – Child stimuli | 734 | 676 | / | / | 120.0135 | 110.9039 | 113.3865 | 0.5115 | 0.9778 | 0.5001 | 0.3767 |
| Viewing time PCSO – Adult vs. child stimuli | 987 | 734 | 32.8 | / | 103.7337 | 120.0135 | 112.1643 | 2.2556 | 0.9578 | 2.1603 | 0.5627 |

Note: *M*_1,_ *SD*_1,_ SE_1_= mean, standard deviation, and standard error of PCSO. *M*_2,_ *SD*_2,_ SE_2_ *= mean, standard deviation, and standard error of the control sample.* Child stimuli: mean is calculated for boy + girl child stimuli, standard deviation is calculated for boy + girl child stimuli (= *Pooled SD*). Formula’s used: ***Pooled SD*** = √[(*n*_1_ - 1)**SD*_1_^2^ + (*n*_2_ - 1)**SD*_2_^2^] / (*n*_1_ + *n*_2_ - 2). ***g*** = (*M*_1_ - *M*_2_) / √[(*n*_1_ - 1)**SD*_1_^2^ + (*n*_2_ - 1)**SD*_2_^2^] / (*n*_1_ + *n*_2_ - 2). Since N < 50 (*n*_1_ PCSO = 10 + *n*_2_ HC = 26), g was multiplied by a correction **factor J** = 1-3/(4*N*-9). **SE_g_** = √[(*n*_1_ + *n*_2_) / *n*_1_**n*_2_ + *g*^2^ / 2(*n*_1_ + *n*_2_)].

**Supplementary Table 3: List of Raw Data Vásquez-Amézquita et al. (2019)**

|  | ***M*_1_** | ***M*_2_** | ***SD*_1_** | ***SD*_2_** | ***Pooled SD*** | **Hedges’ g** | **Factor J** | **Hedges’ g (corrected)** | **SE_g_** |
| --- | --- | --- | --- | --- | --- | --- | --- | --- | --- |
| Viewing time PCSO vs. HC – Adult stimuli | 1.115 | 1.3 | 0.5756 | 0.8146 | 0.7087 | -0.2611 | 0.9784 | -0.2555 | 0.3303 |
| Viewing time PCSO vs. HC – Child stimuli | 0.795 | 0.65 | 0.42 | 0.4504 | 0.4359 | 0.3326 | 0.9784 | 0.3254 | 0.3312 |
| Viewing time PCSO vs. SOAA – Adult stimuli | 1.115 | 0.865 | 0.5756 | 0.5316 | 0.5555 | 0.4500 | 0.9764 | 0.4394 | 0.3477 |
| Viewing time PCSO vs. SOAA – Child stimuli | 0.795 | 0.445 | 0.42 | 0.3905 | 0.4064 | 0.8612 | 0.9764 | 0.8409 | 0.3584 |
| Viewing time PCSO vs. Offenders – Adult stimuli | 1.115 | 0.7618 | 0.5756 | 0.5436 | 0.5547 | 0.6368 | / | / | 0.2981 |
| Viewing time PCSO vs. Offenders – Child stimuli | 0.795 | 0.4503 | 0.42 | 0.4029 | 0.4088 | 0.8432 | / | / | 0.3030 |
| Fixation latency PCSO vs. HC – Adult stimuli | 1.10 | 0.94 | 0.2750 | 0.3206 | 0.2993 | 0.5345 | 0.9784 | 0.5230 | 0.3595 |
| Fixation latency PCSO vs. HC – Child stimuli | 1.02 | 1 | 0.2750 | 0.2761 | 0.2756 | 0.0726 | 0.9784 | 0.0710 | 0.3290 |
| Fixation latency PCSO vs. SOAA – Adult stimuli | 1.10 | 1.245 | 0.2750 | 0.4950 | 0.4004 | -0.3683 | 0.9764 | -0.3596 | 0.3408 |
| Fixation latency PCSO vs. SOAA – Child stimuli | 1.02 | 1.23 | 0.2750 | 0.4153 | 0.3479 | -0.6036 | 0.9764 | -0.5894 | 0.3509 |
| Fixation latency PCSO vs. Offenders – Child stimuli | 1.02 | 1.2538 | 0.2750 | 0.4227 | 0.3790 | -0.6169 | / | / | 0.2977 |
| Viewing time PCSO – Adult vs. child stimuli | 1.115 | 0.795 | 0.5756 | 0.42 | 0.5038 | 0.6352 | 0.9778 | 0.6211 | 0.3413 |
| Fixation latency PCSO – Adult vs. child stimuli | 1.10 | 1.02 | 0.2750 | 0.2750 | 0.2750 | 0.2909 | 0.9778 | 0.2844 | 0.3350 |

Note: *M*_1,_ *Pooled SD*_1=_ mean and pooled standard deviation of male + female stimuli for PCSO. *M*_2,_ *Pooled SD*_2_ *=* mean and pooled standard deviation of male + female stimuli for control sample. *Pooled SD* = Pooled standard deviation of pooled *SD_1_* and *SD_2._* Formula’s used: ***Pooled SD*** = √[(*n*_1_ - 1)**SD*_1_^2^ + (*n*_2_ - 1)**SD*_2_^2^] / (*n*_1_ + *n*_2_ - 2). ***g*** = (*M*_1_ - *M*_2_) / √[(*n*_1_ - 1)**SD*_1_^2^ + (*n*_2_ - 1)**SD*_2_^2^] / (*n*_1_ + *n*_2_ - 2). When N < 50 (*n*_1 +_ *n*_2_ < 50), g was multiplied by a correction **factor J** = 1-3/(4*N*-9). **SE_g_** = √[(*n*_1_ + *n*_2_) / *n*_1_**n*_2_ + *g*^2^ / 2(*n*_1_ + *n*_2_)]. *n*PCSO = 18, *n*SOAA = 16, *n*HC = 19, *n*Offenders = 34 (SOAA + NSO).

**Supplementary Table 4: List of Raw Data Fromberger et al. (2012)**

|  | ***t*** | **Hedges’ g** | | **Factor J** | **Hedges’ g (corrected)** | **SE_g_** | |
| --- | --- | --- | --- | --- | --- | --- | --- |
| Fixation latency PCSO vs. HC – Child stimuli | 2.29 | -0.5824 | | / | / | 0.2588 | |
| Fixation latency PCSO vs. SOAA – Child stimuli | 3.190 | -1.3170 | | 0.973 | -1.2814 | 0.4448 | |
|  | ***MD*** | ***SE*** | ***SD*** | **Hedges’ g** | **Factor J** | **Hedges’ g (corrected)** | **SE_g_** |
| Viewing time PCSO – Adult vs. child stimuli | 225.25 | 381.71 | 1790.3786 | -0,0275 | 0.9820 | -0,027 | 0.3015 |
| Fixation latency PCSO – Adult vs. child stimuli | 111.17 | 45.65 | 214.1174 | -0,1133 | 0.9820 | -0,1113 | 0.3017 |

Note: Formulas used: ***g*** = *t* {[√(*n*_1_ + *n*_2_)] / (√*n*_1_*n*_2_)}. When N < 50 (*n*_1 +_ *n*_2_ < 50), g was multiplied by a correction **factor J** = 1-3/(4*N*-9). **SE_g_** = √[(*n*_1_ + *n*_2_) / *n*_1_**n*_2_ + *g*^2^ / 2(*n*_1_ + *n*_2_)]. *n*PCSO = 22, *n*SOAA = 8, *n*HC = 52.

**Supplementary Table 5: List of Raw Data Kamenskov et al. (2020)**

|  | ***M*_1_** | ***M*_2_** | ***SD*_1_** | ***SD*_2_** | ***Pooled SD*** | **Hedges’ g** | **Factor J** | **Hedges’ g (corrected)** | **SE_g_** |
| --- | --- | --- | --- | --- | --- | --- | --- | --- | --- |
| Viewing time PCSO vs. HC – Adult stimuli | 12 448 | 11 556 | 205 | 196 | 200.55 | 4.4621 | / | / | 0.3746 |
| Viewing time PCSO vs. HC – Child stimuli | 11 872 | 10 659 | 280 | 170 | 231.6247 | 5.4185 | / | / | 0.4331 |
| Fixation Latency PCSO vs. HC – Adult stimuli | 396 | 659 | 58 | 77 | 68.1652 | -3.7844 | / | / | 0.3353 |
| Fixation Latency PCSO vs. HC – Child stimuli | 627 | 731 | 114 | 64 | 92.446 | -1.1693 | / | / | 0.2183 |
| Viewing time PCSO – Adult vs. child stimuli | 12 448 | 11 872 | 205 | 280 | 245.3824 | 2.3474 | / | / | 0.2803 |
| Fixation latency PCSO – Adult vs. child stimuli | 396 | 627 | 58 | 114 | 90.4436 | -2.5541 | / | / | 0.2906 |

Note: *M*_1,_ *SD*_1_= mean and standard deviation of PCSO. *M*_2,_ *SD*_2_ *= mean and standard deviation of the control sample.* *Formulas used:* ***Pooled SD*** = √[(*n*_1_ - 1)**SD*_1_^2^ + (*n*_2_ - 1)**SD*_2_^2^] / (*n*_1_ + *n*_2_ - 2). ***g*** = (*M*_1_ - *M*_2_) / √[(*n*_1_ - 1)**SD*_1_^2^ + (*n*_2_ - 1)**SD*_2_^2^] / (*n*_1_ + *n*_2_ - 2). **SE_g_** = √[(*n*_1_ + *n*_2_) / *n*_1_**n*_2_ + *g*^2^ / 2(*n*_1_ + *n*_2_)].
